# Synthetic data-driven AI approach for fetal chromosomal aneuploidies detection

**DOI:** 10.1093/bioadv/vbaf244

**Published:** 2025-10-06

**Authors:** Changhoe Hwang, Krishna Prasad Adhikari, Gyeongin Oh, Sunshin Kim

**Affiliations:** Department of Research & Development, Genomecare Inc., Suwon, Gyeonggi, 16229, Korea; Department of Research & Development, Genomecare Inc., Suwon, Gyeonggi, 16229, Korea; Department of Research & Development, Genomecare Inc., Suwon, Gyeonggi, 16229, Korea; Department of Research & Development, Genomecare Inc., Suwon, Gyeonggi, 16229, Korea

## Abstract

**Motivation:**

A major limitation in the development of fetal chromosomal aneuploidy detection technologies lies in the scarcity of real positive data. To address this issue, we propose a novel methodology to generate virtually unlimited synthetic negative and positive datasets with >99.9% similarity to real data, enabling accurate detection of both autosomal chromosome aneuploidies (ACA) and sex chromosome aneuploidies (SCA). In terms of methods, blood samples from 15 999 pregnant women were analyzed, including 186 clinically confirmed positive cases. Using 701 high-confidence negatives as a reference, we designed algorithms for synthetic data generation. For negatives, multiple real FASTQ files were randomly merged, and fetal fraction (FF) was recalculated to reflect biological variability. For positives, chromosome-specific read counts were adjusted using numerical equations: ACAs were simulated by increasing the target chromosome reads, and SCAs were generated by adjusting sex chromosome read counts using regression models that account for FF and total read count, with the GC distribution preserved. Logistic regression (LR) models were then trained using features including FF, GC content, and chromosomal read counts. Performance was evaluated against conventional *z*-score methods and real positive cases.

**Results:**

From high-confidence negative samples, ∼160 000 synthetic training datasets were generated for major ACA and ∼35 000 for each SCA. While *z*-score methods showed declines in sensitivity (T13) or positive predictive value (PPV) (T18, T21) under low prevalence, LR models consistently maintained 100% sensitivity and PPV for ACAs, achieved ≥99.6% sensitivity and PPV for SCAs on synthetic evaluation datasets, and demonstrated 100% accuracy on real positive samples.

**Availability and implementation:**

All formulas and procedures required for synthetic data generation and model development are implemented in Python and are available at https://github.com/genomecare-rnd/SyntheticData-NIPT.

## 1 Introduction

Fetal chromosomal aneuploidy is defined as numerical chromosomal abnormalities in the fetus. It includes autosomal chromosome aneuploidies (ACA) such as trisomy 13 (T13), trisomy 18 (T18), and trisomy 21 (T21), as well as sex chromosome aneuploidies (SCA), and represents the target of non-invasive prenatal testing (NIPT). To detect ACA and SCA, NIPT based on cell-free DNA (cfDNA) fragments obtained via liquid biopsy has gained increasing demand among pregnant women and clinicians due to its safety and convenience. However, substantial improvements are still required in terms of sensitivity and positive predictive value (PPV). In particular, PPV remains relatively low, ranging from 30% to 90%. For instance, Bianchi *et al.* (2014) reported an average PPV of 42.75% for sequencing-based screening of T18 and T21, while Zhang *et al.* (2015) and Norton *et al.* (2015) reported average PPVs of 85.27% and 69.6% for T13, T18, and T21, respectively. Additionally, Taylor-Phillips *et al.* (2016) concluded that the average PPV of NIPT for major trisomies remains suboptimal at approximately 71.6%. Such limitations have also been observed in the previously cited studies, which employed statistical approaches including *z*-score-based methods.

The insufficient accuracy of NIPT results often leads to unnecessary invasive diagnostic procedures such as amniocentesis or chorionic villus sampling, increasing both the physical and psychological burden on pregnant women. Moreover, misinterpretation of test results may result in termination of pregnancies that are, in fact, chromosomally normal (Liao *et al.* 2014). Therefore, enhancing the accuracy and reliability of NIPT is crucial to minimize false-positive outcomes.

A major limitation in improving NIPT accuracy lies in the scarcity of real positive samples with genetic abnormalities. Since NIPT relies on distinguishing between positive and negative datasets, the rarity of real positives hinders the definition of clear classification boundaries. Furthermore, optimal threshold values differ depending on the specific chromosomal abnormality being screened, underscoring the need for diverse and abundant positive datasets to enable technological advancements. However, the persistent scarcity of such data remains a fundamental limitation.

To address these challenges, we propose a novel approach to generate virtually unlimited synthetic negative and positive datasets with over 99.9% similarity to real-world data, enabling accurate detection of both ACA and SCA. This study presents the methodology and evaluation results of the proposed approach. Our framework leverages artificial intelligence (AI), including machine learning techniques, particularly logistic regression (LR) models (Hosmer *et al.* 2013), to robustly classify chromosomal abnormalities using synthetic data. Unlike conventional statistical approaches, LR models can integrate multiple genomic features simultaneously, capture complex patterns in the data, and effectively leverage large-scale synthetic datasets. These advantages enable the improvement of sensitivity and PPV in the detection of fetal chromosomal aneuploidy. To objectively assess the accuracy of our technique, model performance was evaluated across varying prevalence rates, considering that the PPV of NIPT tends to be elevated in high-risk populations and diminished in low-risk populations (American College of Obstetricians and Gynecologists 2015).

## 2 Methods

**Figure 1.**
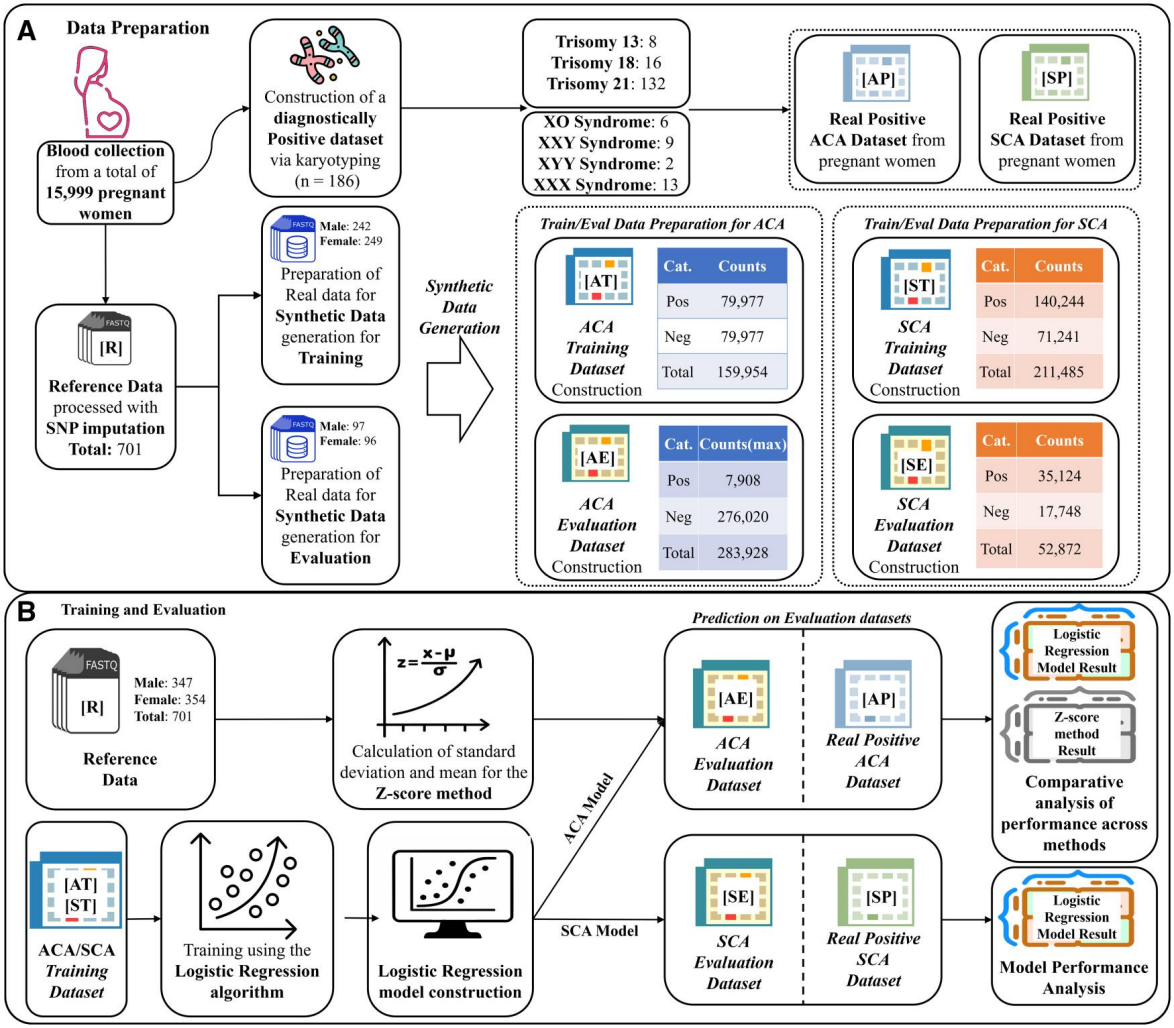
Overview of the research workflow. Figure 1A shows the schematic process of synthetic data generation in this study, and Figure 1B presents the workflow of model training and evaluation using the generated data.

Figure 1 summarizes the research methodology. In Section A, blood samples were initially collected from 15 999 pregnant women. To identify actual ACA and SCA cases, karyotyping was performed on the collected samples. Through this process, 186 clinically confirmed positive cases were selected as the real positive dataset. These are denoted as [AP]/[SP].

In the next step, the maternal samples were sequenced using a single-nucleotide polymorphism (SNP) imputation approach, which applies probabilistic inference of unobserved genotypes based on linkage disequilibrium patterns and reference panels to enhance the resolution of genetic data (Kim *et al.* 2018). After applying filtering criteria, 701 high-confidence negative fetal samples were selected as the reference dataset [R].

The reference dataset was partitioned to generate training and evaluation datasets for each type of aneuploidy. We constructed the datasets using the synthetic data generation algorithm proposed in this study. For both ACA and SCA, the training datasets—denoted as [AT] and [ST]—were configured to maintain 34 000∼36 000 synthetic data across all labels.

The ACA evaluation dataset [AE] was constructed using prevalence rates reported in Presson *et al.* (2013) and Goel *et al.* (2019), with upscaling applied accordingly. The evaluation dataset for SCA [SE] was generated with approximately 8535–9094 samples per sex chromosome condition.

Section B provides a summary of the training and evaluation process for chromosomal aneuploidy classification models. These models were developed as machine learning models based on the LR algorithm and, in this paper, are collectively referred to as the LR model. We employed the LR model to develop high-accuracy classification models for ACA and SCA using synthetic training data. The training datasets [AT] and [ST] were used for ACA and SCA model training. Features used for model development included fetal fraction (FF), GC content, and chromosomal read counts.

To evaluate the performance of the models, different evaluation strategies were applied for ACA and SCA detection. For the evaluation of ACA, a comparative analysis was conducted between the outputs of the LR-based ACA model and those obtained using the conventional *z*-score method. To enable this comparison, a *z*-score classifier was constructed using the reference dataset [R] to determine the presence or absence of ACA in the input data, and it was applied to both the ACA evaluation dataset [AE] and the actual ACA cases [AP], providing a consistent basis for assessing the performance of the LR-based model against the conventional approach. To evaluate SCA detection, model performance was assessed based on the prediction results obtained from the LR SCA model using both [SE] and [SP]. By analyzing the classification performance derived from the above procedures, we ultimately aimed to validate the utility of the synthetic data generation methodology proposed in this study.

### 2.1 Sample preparation

We collected blood samples from 15 999 pregnant women. Among the collected samples, amniocentesis was performed for those identified as positive in order to conduct fetal karyotyping. As a result, the following cases of chromosomal aneuploidies were confirmed in the real positive dataset: 8 cases of T13, 16 cases of T18, 132 cases of T21, 6 cases of XO, 9 cases of XXY, 2 cases of XYY, and 13 cases of XXX.

### 2.2 Preparation of cell-free DNA and DNA library for sequencing

Approximately 10 ml of peripheral blood was collected from each subject using BCT™ tubes (Streck, Omaha, NE, USA). The collected blood samples were centrifuged at 1200 × *g* for 15 min at 4°C. After plasma separation, it was transferred to new tubes and centrifuged again at 16 000 × *g* for 10 min at the same temperature. cfDNA was extracted from the centrifuged plasma using the MagListo™ cfDNA Extraction Kit (Bioneer, Korea).

The extracted cfDNA fragments were end-repaired using T4 DNA polymerase, Klenow DNA polymerase, and T4 polynucleotide kinase, followed by purification of high-quality cfDNA fragments using Agencourt AMPure XP (Beckman Coulter, USA).

DNA libraries for Ion Proton sequencing were prepared according to the protocol provided by Thermo Fisher Scientific. An average sequencing coverage depth of 0.3× per nucleotide was achieved using the 540 chip.

### 2.3 High-throughput parallel sequencing

High-throughput parallel sequencing was performed using the Ion Torrent S5™ XL system (Thermo Fisher Scientific) with the prepared DNA libraries. Raw sequencing reads were obtained using the Ion Torrent Suite™ software (Thermo Fisher Scientific). The filtered reads were then aligned using the Burrows-Wheeler transform algorithm (Manzini 2001). Sequence reads that mapped to a single unique genomic location in hg19 were defined as unique reads. On average, approximately 3.3 × 10^6^ reads were identified as unique reads from the total sequencing reads, and the GC content of the 15 999 datasets ranged from approximately 39%–45%.

## 3 Algorithm

### 3.1 Algorithm for generating synthetic negative data from real negative data

The first step in generating synthetic negative data is to prepare real negative data. The real negative data were used to estimate FFs through the SNP imputation technique (Kim *et al.* 2018). These data were categorized into male and female fetal groups, and all were provided in the form of raw FASTQ files.

The next step is to randomly select two or more nonoverlapping FASTQ files from either the male or female fetal group. The selected FASTQ files are combined into a single unit by merging their read information—including sequence IDs, sequences, “+,” and quality scores—and then randomly shuffled to generate a new synthetic FASTQ file with a desired number of reads (e.g. 2 million or 3 million reads).

For example, in the context of FF estimation, if FASTQ file A contains A reads with an FF of $A_{ff}$, and FASTQ file B contains B reads with an FF of $B_{ff}$. Then, the overall FF of the combined FASTQ file C can be calculated as follows:


$$C_{ff}=\frac{A\cdot A_{ff}\;+\;B\cdot B_{ff}}{A\;+\;B}$$

Therefore, a newly generated FASTQ file, created by randomly sampling reads from the combined FASTQ file C, will retain the same FF as C.

When mixing two or more FASTQ files at random, the FF is calculated using the following formula:


$$\frac{{(M}_{1}\cdot M_{1ff}\;+\;\cdots \;+\;M_{i}\cdot M_{\mathrm{iff}}\;+\;\cdots \;+\;M_{n}\cdot M_{\mathrm{nff})}}{M_{1}\;+\;\cdots \;+\;M_{i}\;+\;\cdots \;+\;M_{n}}$$

In the formula above, $M_{i}$ represents the number of reads in the $i^{th}$ FASTQ file, and $M_{\mathrm{iff}}$ denotes the FF of $M_{i}$, where i ranges from 1 to *n*.

To evaluate the similarity between real and synthetic data, frequency distributions of DNA fragment sizes were calculated for real and synthetic sample sets (*n* = 20 each) at FFs of 5%, 10%, and 15%, respectively. Correlation coefficients between the distributions were then computed to assess their similarity. As shown in Table 1 and Fig. 1, available as supplementary data at *Bioinformatics Advances* online, all correlation coefficients exceeded 99.9%, demonstrating a high degree of similarity between the real and synthetic data.

**Table 1.** Correlation of read length frequency distributions between real and synthetic data.

Fetal fraction (%)	Correlation coefficient
5	99.9983
10	99.9929
15	99.9931

### 3.2 Algorithm for generating synthetic positive data from synthetic negative data

When the target chromosome is an autosome, the process of generating synthetic positive data from synthetic negative data can be expressed by the following equation:


(1) $$T_{i}RC=C_{i}RC+\frac{C_{i}RC\times FF}{2}$$

In Equation (1), $T_{i}RC$ represents the read count of the target chromosome i in the presence of trisomy, $C_{i}RC$ denotes the read count of the same chromosome in a normal fetus.

When the target chromosome is XXX, the read count in the synthetic positive data is calculated using the equation below:


(2) $$C_{\mathrm{XXX}}RC=C_{XX}RC+\frac{C_{XX}RC\times FF}{2}$$

In Equation (2), $C_{\mathrm{XXX}}RC$ represents the read count of the XXX chromosome in a fetus with XXX syndrome, $C_{XX}RC$ denotes the read count of the XX chromosome in a normal female fetus.

When the target chromosome is XO, the read count is determined by the following equation:


(3) $$C_{XO}RC=C_{XY}RC-C_{Y}RC+C_{y}RC$$

In Equation (3), $C_{XO}RC$ represents the read count of the X chromosome in a fetus with XO syndrome, $C_{XY}RC$ is the read count of the XY chromosome in a normal male fetus, $C_{Y}RC$ denotes the read count assigned to the Y chromosome in a normal male fetus, and $C_{y}RC$ indicates the number of reads erroneously assigned to the Y chromosome.

The value of $C_{y}RC$ can be estimated by calculating the residual using the equations as follows:


(3.1) $$C_{y}RC=\beta_{0}+\beta_{1}\cdot \mathrm{TRC}+\varepsilon$$

(3.2) $$C_{y}RC'=\beta_{0}+\beta_{1}\cdot \mathrm{TRC}$$

In Equation (3.1), TRC represents the total read count across all chromosomes, $\beta_{0}$ is the intercept, $\beta_{1}$ is the coefficient between TRC and $C_{y}RC$, and ε denotes the residual. In Equation (3.2), $C_{y}RC$′ represents the predicted value of $C_{y}RC$.

Theoretically, female fetuses (XX) should have no reads assigned to the Y chromosome. However, due to read misalignment, some reads may be erroneously assigned to the Y chromosome, and this misassignment tends to increase proportionally with TRC. Therefore, a model can be constructed using Equations (3.1) and (3.2) based on female fetal data.

This model can also be approximately applied to male fetal (XY) data, based on the assumption that the number of misassigned reads to the Y chromosome increases proportionally with TRC. In other words, the TRC of a female fetus can be approximated as the TRC of a male fetus. The distribution of the residuals is assumed to follow a normal distribution, characterized by a specific standard deviation. By calculating $C_{y}RC$′ using Equation (3.2) and adding a randomly generated residual from a normal distribution with the same mean and standard deviation observed in female fetal data, the misassigned $C_{y}RC$ in male fetal data can be approximated.

When the target SCA is XYY, the equation is given as follows:


(4) $$C_{\mathrm{XYY}}RC=C_{XY}RC+C_{Y}RC-C_{y}RC$$

In Equation (4), $C_{\mathrm{XYY}}RC$ represents the read count of the XYY chromosome in a fetus with XYY syndrome, and $C_{XY}RC$ denotes the read count of the XY chromosome in a normal male fetus.

When the target is XXY, the read count in the synthetic positive data can be calculated using the equation below:


(5) $$C_{\mathrm{XXY}}RC=C_{XX}RC+C_{Y}RC$$

In the equation above, $C_{\mathrm{XXY}}RC$ represents the read count of the XXY chromosome in a fetus with XXY syndrome. $C_{XX}RC$ denotes the read count of the XX chromosome in a normal female fetus, and $C_{Y}RC$ indicates the read count assigned to the Y chromosome. The value of $C_{Y}RC$ can be calculated using the following equation:


(5.1) $$C_{Y}RC=\beta_{0}+\beta_{1}\cdot \mathrm{TRC}+\beta_{2}\cdot {FF}_{\mathrm{snp}}+\varepsilon$$

(5.2) $$C_{Y}RC'=\beta_{0}+\beta_{1}\cdot \mathrm{TRC}+\beta_{2}\cdot {FF}_{\mathrm{snp}}$$

In Equation (5.1), TRC represents the total read count across all chromosomes, ${FF}_{\mathrm{snp}}$ is the FF estimated from SNPs, and $\beta_{0}$ is the intercept. $\beta_{1}$ is the coefficient between the total read count and the Y chromosome read count, while $\beta_{2}$ is the coefficient between the FF and the Y chromosome read count. ε denotes the residual, and $C_{Y}RC$′ represents the predicted Y chromosome read count.

This regression model was constructed using data from normal male fetuses (XY). The residuals are assumed to follow a normal distribution, and the XXY syndrome data are expected to exhibit a regression pattern and residual distribution similar to those of normal male fetal data.

Because $C_{Y}RC$ is very small compared to TRC, the TRC and SNP-based FF from normal female (XX) fetal data can be used as proxy values for the XXY syndrome data. By calculating $C_{Y}RC\prime$ using Equation (5.2) and adding a randomly sampled residual from a normal distribution with the same mean and standard deviation as that of the residuals in normal male fetal data, the Y chromosome read count in female fetal data can be approximately estimated.

### 3.3 Artificial intelligence models

ACA classification models were developed using synthetic normal and synthetic positive data. The models utilize $C_{i}RC$, along with the FF and GC content, as input features. For SCA classification models, input features include $C_{X}RC$, $C_{Y}RC$, FF, and GC content. Models were trained using the LR machine learning algorithm optimized for the detection of chromosomal aneuploidies.

In this study, we adopted LR as the classification algorithm, owing to its simplicity, interpretability, and computational efficiency, rather than relying on other machine learning approaches that demand more complex computations. For ACA, LR was applied in a binary classification setting, distinguishing between normal and abnormal cases. SCA, LR was extended to a multi-class framework, enabling the simultaneous discrimination of multiple categories of abnormalities.

LR models can incorporate heterogeneous genomic features, including chromosomal read count, FF, and GC content, without requiring extensive preprocessing. Furthermore, the coefficient structure of LR allows for straightforward interpretation of feature contributions, ensuring transparency and reliability in diagnostic decision-making. Finally, LR provides a robust yet efficient solution for fetal chromosomal aneuploidy classification without the need for more computationally intensive machine learning algorithms.

To improve classification accuracy, the total read count for each sample was normalized to a baseline of 3 million reads. This normalization fixes the total read count of each sample to a constant value, allowing the model to be trained based on variations in read counts across each chromosome and enabling robust detection of aneuploidies.

The algorithmic flow of synthetic data generation and machine learning model training process is illustrated in Fig. 14, available as supplementary data at *Bioinformatics Advances* online, and its implementation is available at https://github.com/genomecare-rnd/SyntheticData-NIPT.

## 4 Results

Out of the 15 999 maternal samples collected, 347 male and 354 female fetal samples that satisfied the inclusion criteria were selected. The criteria included: at least 2 million unique reads, GC content between 39.5% and 43%, and an FF of 4% or higher. For the selected samples, FFs were estimated using SNP genotyping and imputation methods. A minor allele frequency filtering threshold of 7% was applied, and the correlation coefficient between SNP-based FF and Y chromosome-based FF was 98% (Kim *et al.* 2018).

To generate synthetic data, the algorithm described in Sections 3.1 and 3.2 was applied. In detail, two or three real male or female fetal samples were randomly combined from the 347 male and 354 female fetal datasets to generate new synthetic negative and positive data for male and female fetuses. This algorithm enabled systematic generation of synthetic data while preserving biological variability.

### 4.1 Generation and analysis of synthetic data for ACA

To develop classification models for ACA, each sample was represented using $C_{i}RC$, FF, and GC content as input features. The total read count for each sample was fixed at 3 million reads for normalization. When targeting ACA, 160 000 synthetic training samples—comprising 80 000 negatives and 80 000 positives—were generated using 242 real normal male fetal samples and 249 real normal female fetal samples. After removing statistical outliers from the 160 000 synthetic training samples, a total of 159 954 samples were used for model training (Figs 3 and 4, available as supplementary data at *Bioinformatics Advances* online).

For evaluation data generation, positive and negative synthetic samples were created by randomly combining 2–3 samples from 97 real normal male fetal datasets and 96 real normal female fetal datasets that were not used in the training data generation. The selected samples had more than 2 million unique reads, GC content between 39.5% and 43%, and an FF of at least 4%. FFs for these samples were estimated using SNP genotyping and imputation methods.

#### 4.1.1 Trisomy 13

In normal fetuses, the DNA reads assigned to chromosome 13 originate from two copies of maternal chromosomes and two copies of fetal chromosomes. Therefore, the read count for fetal chromosome 13 is determined by multiplying the total reads assigned to chromosome 13 by the FF.

In contrast, in the case of a fetus with T13, an additional fetal chromosome is present, requiring a 50% increase in the fetal DNA read count compared to that of a normal fetus. Consequently, if the number of reads assigned to chromosome 13 in a given dataset is denoted as $C_{13}RC$, then the read count for a fetus with T13, $T_{13}RC$, is calculated using Equation (1) as follows:


$$T_{13}RC=C_{13}RC+\frac{C_{13}RC\;\times \;FF}{2}$$

The same calculation method can be applied to chromosomes 18 and 21 to obtain $T_{18}RC$ and $T_{21}RC$, respectively. The distribution of synthetic positive and negative samples targeting chromosome 13 was visualized in a three-dimensional (3D) space using three key variables—$C_{13}RC$, GC content, and FF—as axes, as shown in Fig. 4, available as supplementary data at *Bioinformatics Advances* online.

To evaluate the performance of the trained model, an evaluation dataset was constructed by statistically incorporating the actual prevalence of T13, T18, and T21. All of the prevalence rates were derived from the studies by Presson *et al.* (2013) and Goel *et al.* (2019).

First, 300 000 synthetic negative samples were generated, and statistical outliers were removed to construct the full dataset. Synthetic positive datasets for T13 were incrementally constructed by upscaling the prevalence from 1× to 40× relative to the synthetic negative dataset. Descriptive statistics for T13 dataset are presented in Table 2.

**Table 2.** Distribution of synthetic data used for training and evaluation of the T13 detection model.

T13 training data		Total training data	Prevalence (T13)
Negative	Positive		
79 977	79 977	159 954	0.55:10000

T13 evaluation data		Total evaluation data
Negative	Positive (upscaled)	
	14 (1×)	268 491
	74 (5×)	268 551
268 477	149 (10×)	268 626
	297 (20×)	268 774
	596 (40×)	269 073

To build the T13 detection model, the training dataset was composed of 79 977 synthetic negative samples and 79 977 synthetic positive samples, as summarized in Table 2. Each sample included three key variables—$C_{13}RC$, GC content, and FF—and the model was trained using an LR algorithm based on these features. To evaluate the model’s performance, evaluation datasets were randomly generated using various upscaling ratios (1×, 5×, 10×, 20×, 40×). As the upscaling ratio decreased, the number of positive samples approached the actual prevalence, allowing for the evaluation of the model’s sensitivity and PPV.

**Figure 2.**
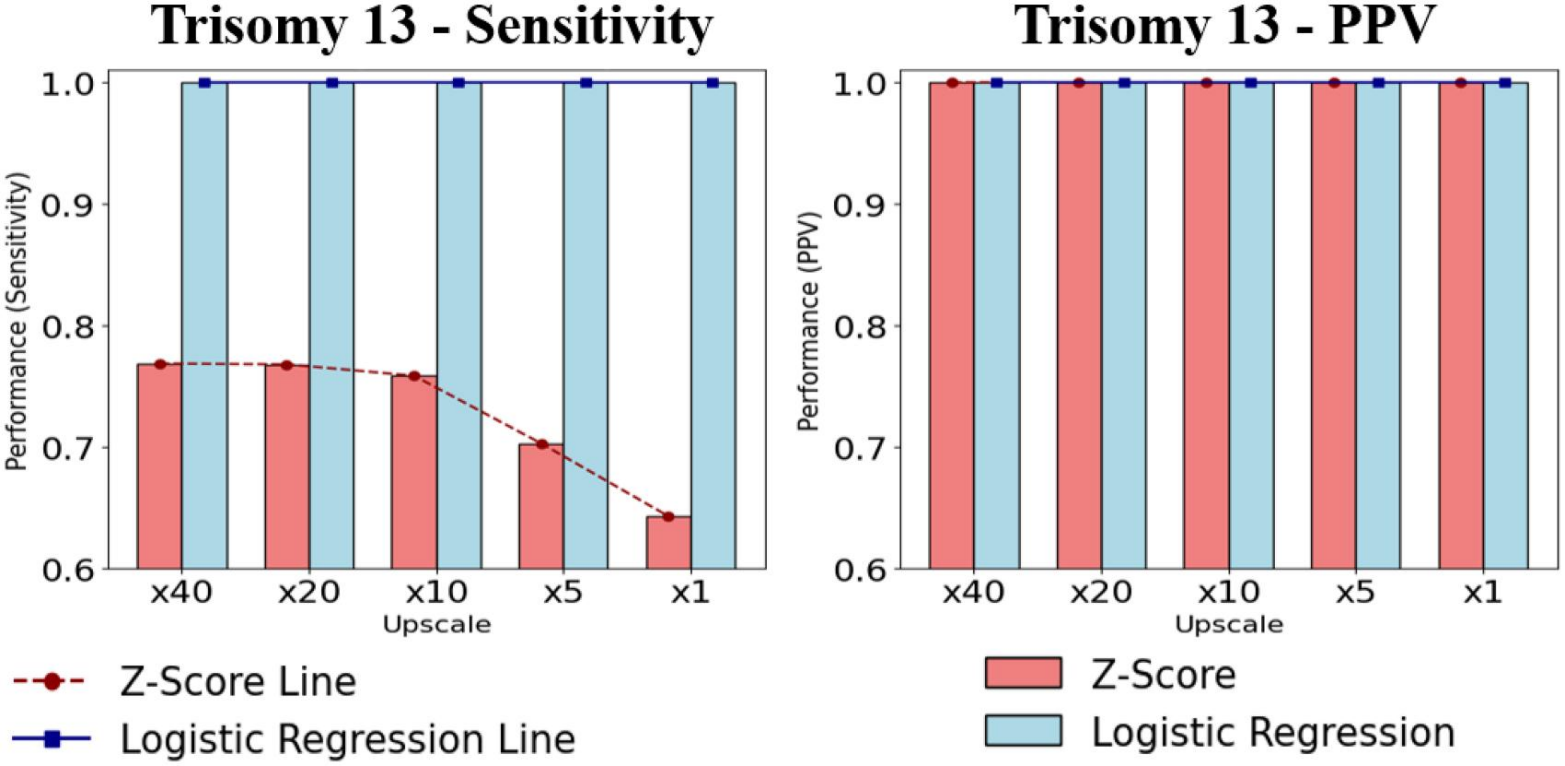
Performance comparison of T13 detection across upscaling factors.

Additionally, to enable a clearer comparison of model performance, detection results from a *z*-score classifier—applied to the outlier-filtered subset of the 701 reference samples—were also analyzed (Liao *et al.* 2014). The presence or absence of ACA was determined using a *z*-score threshold of 3, and the comparison for T13 detection is presented in Fig. 2.

As shown in Fig. 2, both the *z*-score method and the LR model achieved 100% PPV in detecting T13. However, there was a difference in sensitivity. This difference is attributed to the relatively strong correlation between GC content and normalized read count in the case of T13 (Fig. 2, available as supplementary data at *Bioinformatics Advances* online), which led to an observed overlap between positive and negative data distributions. Such overlap highlights a key limitation of the *z*-score method. Moreover, as the upscaling ratio approached 1×—meaning the evaluation data distribution became more similar to the actual prevalence—the sensitivity declined from 77% to 64% (Fig. 2 and Fig. 2, available as supplementary data at *Bioinformatics Advances* online). This decline in sensitivity can be interpreted as a result of an increased frequency of false negatives among the positive cases, which emerged as the evaluation dataset more closely reflected the actual prevalence. In contrast, the LR model for T13 consistently maintained a sensitivity of 100% across all evaluation datasets.

An additional analysis comparing the performance of the *z*-score method and LR model for T13 detection using synthetic negative data applying actual prevalence and 8 real positive T13 samples showed the result of 87.5% sensitivity, only representing the limitation of the *z*-score method. This comparative analysis further confirmed the superior performance of the LR model, with detailed results provided in the Fig. 5 and Table 2, available as supplementary data at *Bioinformatics Advances* online. Additionally, in the evaluation using real negative and positive samples, the *z*-score method showed a sensitivity of 87.5% and a PPV of 1.2%, whereas the LR model achieved 100% sensitivity and 80% PPV (Figs 9 and 12, available as supplementary data at *Bioinformatics Advances* online).

#### 4.1.2 Trisomy 18

**Figure 3.**
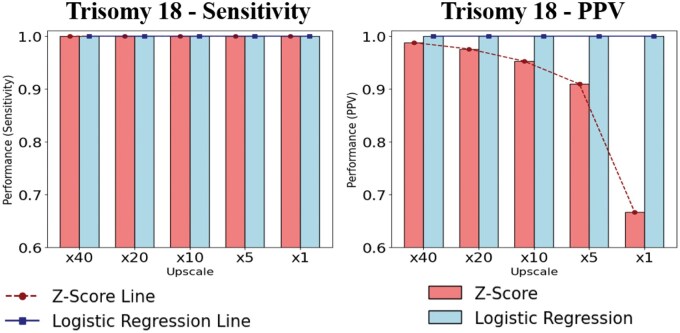
Performance comparison of T18 detection across upscaling factors.

**Figure 4.**
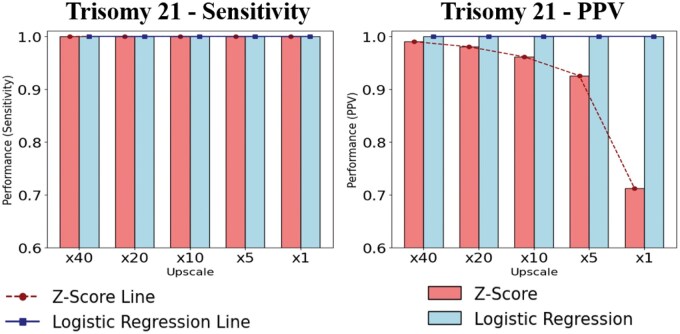
Performance comparison of T21 detection across upscaling factors.

As in Section 4.1.1, we applied the same data generation procedure to T18 to create a balanced set of synthetic negative and positive samples. Subsequently, statistical outliers falling outside the normal range were removed, and optimal synthetic negative and positive datasets were generated for model training (Figs 3 and 4, available as supplementary data at *Bioinformatics Advances* online).

The distribution of synthetic negative and positive data for T18 was visualized in 3D space and is presented in Fig. 4, available as supplementary data at *Bioinformatics Advances* online. These data were randomly mixed and used to train an LR algorithm, through which the T18 detection model was built.

To evaluate T18 detection model’s performance, an evaluation dataset was constructed to statistically reflect the prevalence of T18. Following the same approach as before, synthetic negative data were generated based on actual prevalence, and synthetic T18 data were created by applying upscaling factors ranging from 1 to 40. The descriptive statistics for this dataset are summarized in Table 3.

**Table 3.** Distribution of synthetic data used for training and evaluation of the T18 detection model.

Prevalence^a^	T18 evaluation data		Total evaluation data
	Negative	Positive (upscaled)	
		30 (1×)	276 050
		150 (5×)	276 170
1.07:10000	276 020	299 (10×)	276 319
		597 (20×)	276 617
		1189 (40×)	277 209

a Training data composition is identical to Table 2.

Figure 3 presents the sensitivity and PPV results of the two methods evaluated across each upscaled evaluation dataset. As shown, both the *z*-score method and the LR model achieved 100% sensitivity across all evaluation datasets. However, unlike the T13 evaluation results, the PPV of the T18 *z*-score method decreased from a maximum of 98% to 66% as the evaluation data distribution approached the actual prevalence (Fig. 3 and Fig. 2, available as supplementary data at *Bioinformatics Advances* online). This decline is presumed to result from the increasing proportion of false positives as the prevalence in the evaluation data approaches real-world incidence rates, leading to a statistical rise in the number of negative samples. This is a commonly observed pattern in rare disease detection models, where lower prevalence tends to be associated with reduced PPV.

To support these findings, an additional evaluation was conducted using clinically confirmed T18 cases in combination with the synthetic negative dataset. In this evaluation assessment, the LR model consistently outperformed the *z*-score method across multiple performance metrics. Notably, the *z*-score method exhibited a marked decline in performance under conditions approximating clinical prevalence. This outcome reinforces the pattern observed in the synthetic data evaluation. A comparative analysis is provided in the Fig. 6 and Table 3, available as supplementary data at *Bioinformatics Advances* online.

Moreover, evaluation using real negative and positive samples showed that the *z*-score method achieved 100% sensitivity but only 15% PPV, whereas the LR model demonstrated 100% for both sensitivity and PPV (Figs 10 and 12, available as supplementary data at *Bioinformatics Advances* online).

#### 4.1.3 Trisomy 21

Similarly, for T21, synthetic negative and T21 data were generated, outliers were removed, and 159 954 synthetic samples were used as the training dataset (Figs 3 and 4, available as supplementary data at *Bioinformatics Advances* online). To examine the distribution of the generated T21 training data, a 3D visualization was performed targeting chromosome 21 as shown in Fig. 4, available as supplementary data at *Bioinformatics Advances* online.

To train a T21 detection model using the LR algorithm, randomly mixed synthetic data were used. The performance of the model was evaluated using a dataset consisting of 238 126 synthetic negative samples and up to 7912 synthetic positive samples. The statistical details of this dataset are presented in Table 4.

**Table 4.** Distribution of synthetic data used for training and evaluation of the T21 detection model.

Prevalence^a^	T18 evaluation data		Total evaluation data
	Negative	Positive (upscaled)	
		198 (1×)	238 262
		993 (5×)	239 057
8.27:10000	238 064	1980 (10×)	240 044
		3952 (20×)	242 016
		7908 (40×)	245 972

a Training data composition is identical to Table 2.

Figure 4 presents the performance comparison for T21 detection, showing that the LR model consistently maintained 100% sensitivity and PPV across all upscaling conditions and demonstrated stable performance even at low prevalence levels. In contrast, the *z*-score method showed a decline in PPV from 98.9% to 71%, despite maintaining 100% sensitivity, as the upscaling ratio approached real-world prevalence levels (Fig. 4 and Fig. 2, available as supplementary data at *Bioinformatics Advances* online). This trend is consistent with the general pattern observed in rare disease detection, where the false-positive rate tends to increase as prevalence decreases. These evaluation results using synthetic data were similarly observed when analyzing detection performance on real positive samples and synthetic negative samples according to prevalence (Fig. 7 and Table 4, available as supplementary data at *Bioinformatics Advances* online).

Additionally, in the evaluation using real negative and positive data, the *z*-score method showed 100% sensitivity and 78.1% PPV, whereas the LR model achieved 100% for both sensitivity and PPV (Figs 11 and 12, available as supplementary data at *Bioinformatics Advances* online).

### 4.2 Generation and analysis of synthetic data for SCA

To train SCA classification models, each sample was represented using $C_{X}RC$, $C_{Y}RC$, FF, and GC content as input features. The total read count per sample was fixed to 3 million reads for normalization.

To generate synthetic data for SCA, Equation (2) was used for XXX syndrome. Equation (3) was used for XO, Equation (4) for XYY, and Equation (5) for XXY.

**Figure 5.**
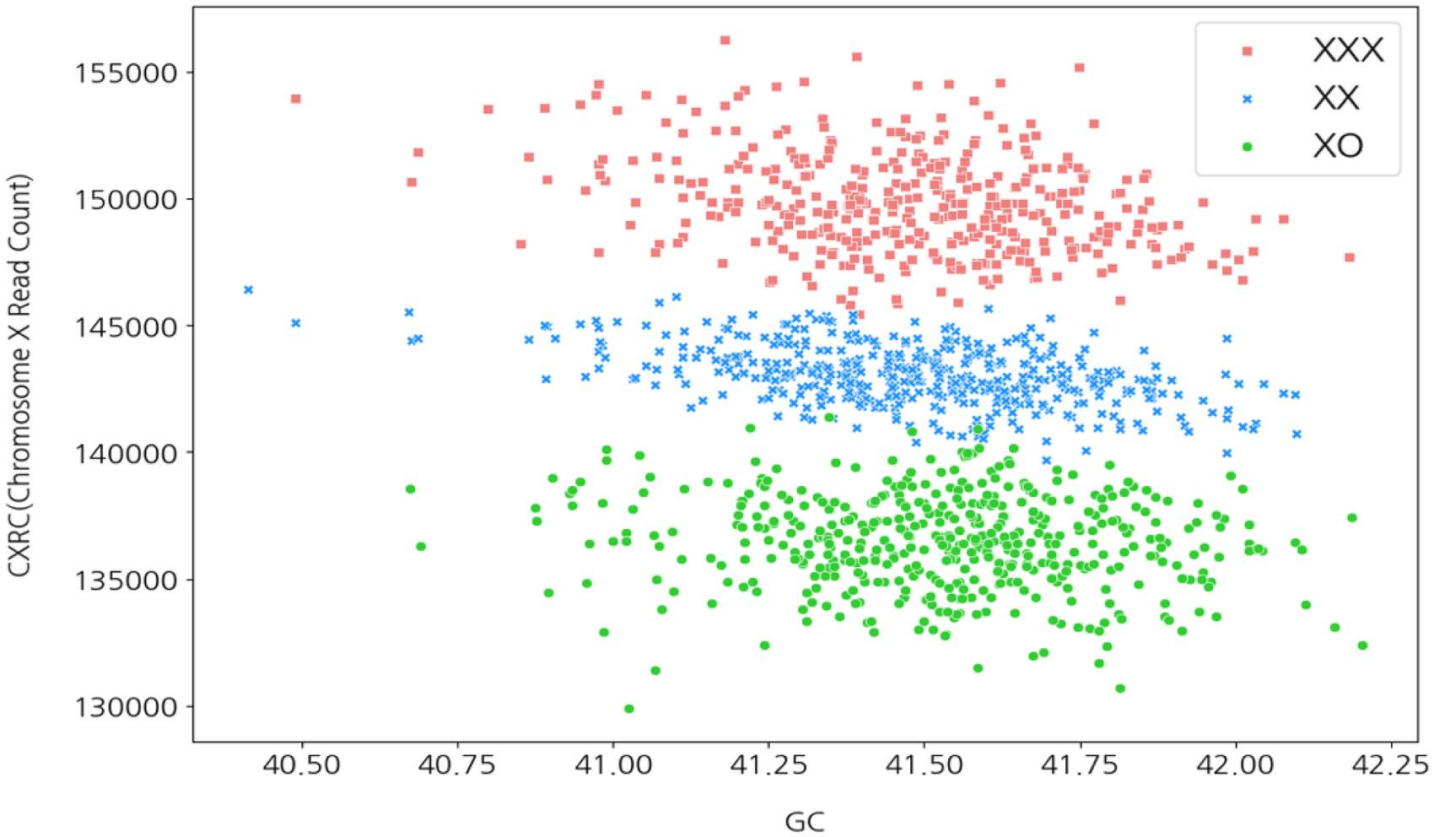
Distribution of positive and negative samples based on $C_{X}RC$ and GC.

Using these equations, synthetic data were generated not only for female fetuses but also for male fetuses, targeting the X chromosome to create both negative (XX, XY) and positive (XO, XXX, XXY) cases. Figure 5 shows the distribution of synthetic negative female (XX) and synthetic positive samples—monosomy X (XO) and triple X syndrome (XXX)—based on two axes: GC content and $C_{X}RC$.

As shown in Fig. 5, for the targeted female X chromosome, the read counts—$C_{XX}RC$, $C_{XO}RC$, and $C_{\mathrm{XXX}}RC$—assigned to $C_{X}RC$ exhibited distinct distributions depending on GC content.

**Figure 6.**
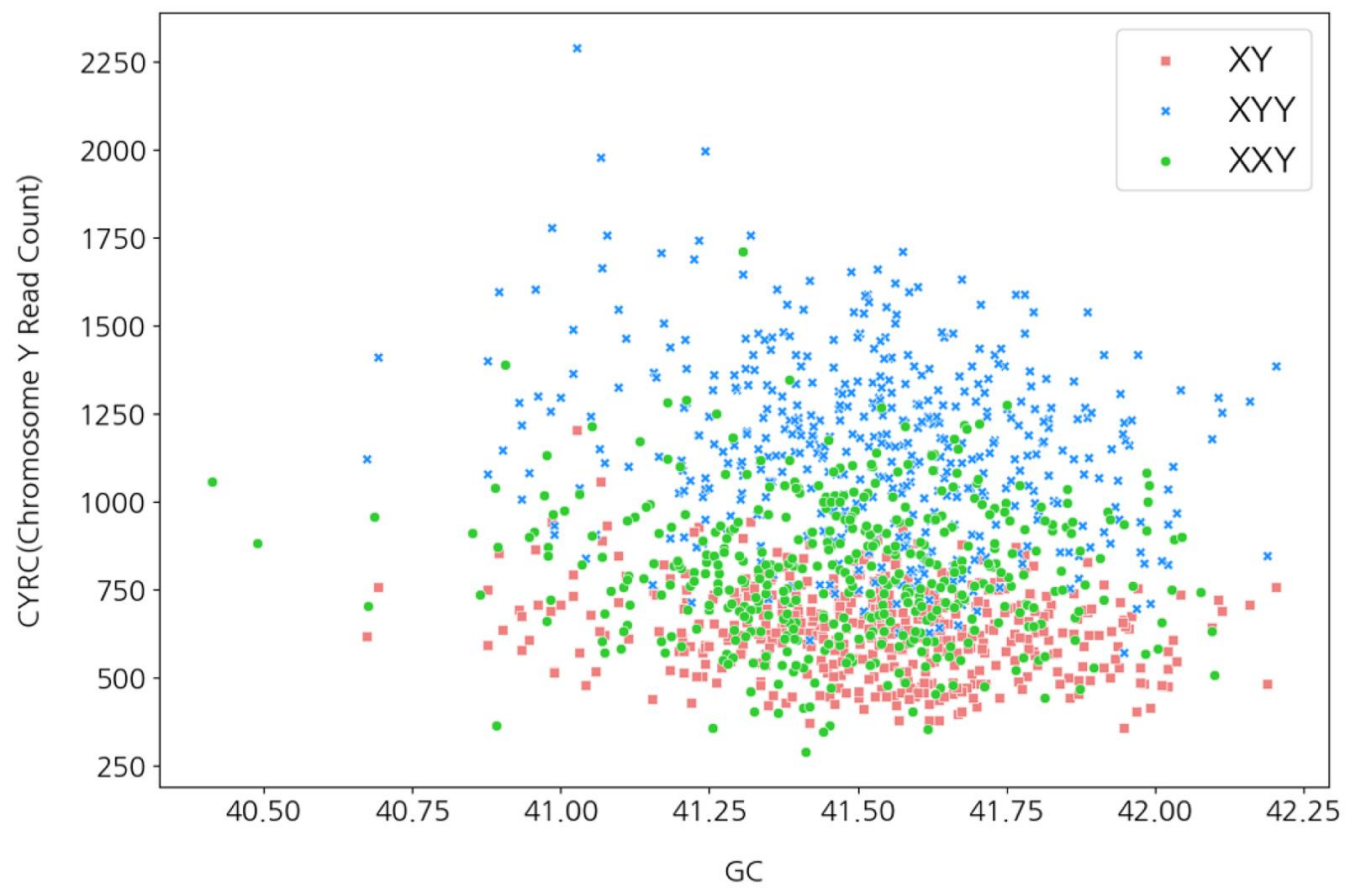
Distribution of positive and negative samples based on $C_{Y}RC$ and GC.

Similarly, Fig. 6 presents the distribution of synthetic normal male fetuses (XY) and synthetic positive cases—XXY and XYY syndromes—targeting the male XY chromosome, based on two axes: GC content and $C_{Y}RC$. For the targeted male Y chromosome, the read counts—$C_{XY}RC$, $C_{\mathrm{XXY}}RC$, and $C_{\mathrm{XYY}}RC$—assigned to $C_{Y}RC$ exhibited distinct distributions depending on GC content.

Distributional differences indicate that $C_{X}RC$, $C_{Y}RC$, FF, and GC content can serve as effective parameters for distinguishing between negative fetal data (XX and XY) and SCA, such as XO, XXX, XXY, and XYY.

To construct training datasets for SCA detection, 242 real normal male fetal samples and 249 real normal female fetal samples were used. After removing outliers, 34 959 synthetic negative XY samples and 36 282 synthetic negative XX samples were generated, along with synthetic positive samples comprising 34 991 XYY, 34 547 XO, 34 602 XXX, and 36 104 XXY cases. To construct the evaluation dataset, synthetic samples were generated using 97 real normal male and 96 real normal female fetal data that were not used in the creation of the training set. The evaluation dataset comprised 8772 synthetic normal XY samples, 8976 synthetic normal XX samples, and synthetic positive samples, including 8740 XYY, 8535 XO, 8755 XXX, and 9094 XXY cases.

**Figure 7.**
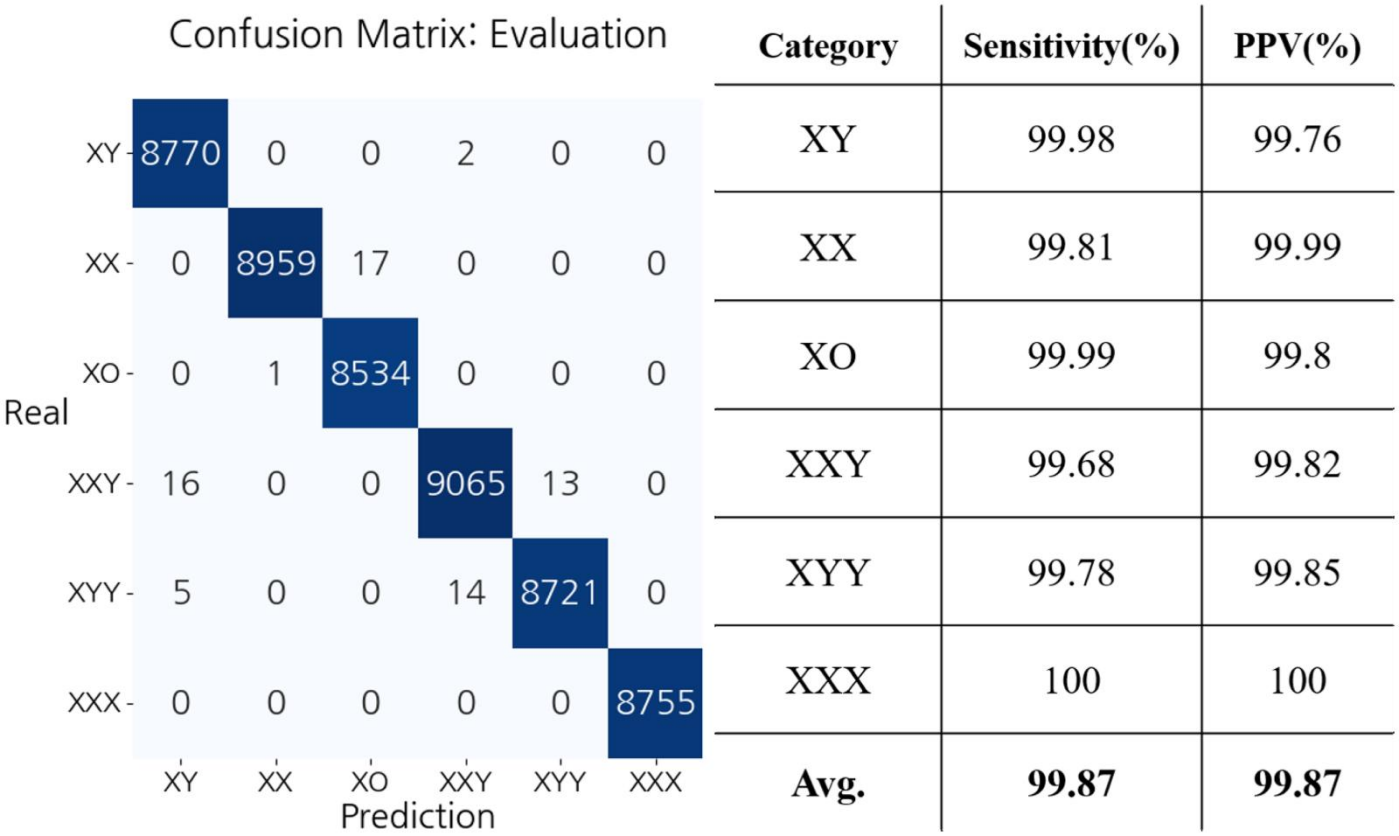
Performance evaluation of SCA detection.

The LR model for SCA classification was implemented using the previously constructed training dataset, and its performance was evaluated using the evaluation dataset. The model achieved over 99.6% accuracy in both sensitivity and PPV on the evaluation dataset, as shown in Fig. 7. Furthermore, the model demonstrated 100% predictive accuracy on real positive and negative samples, confirming the reliability and utility of both the synthetic data and the model (Fig. 13, available as supplementary data at *Bioinformatics Advances* online).

## 5 Discussion

In NIPT, conventional *z*-score screening techniques have inherent limitations in terms of sensitivity and PPV. In particular, the PPV of NIPT tends to remain elevated in high-risk populations but declines significantly in low-risk populations (American College of Obstetricians and Gynecologists 2015). In this study, we evaluated the accuracy of PPV with a focus on both high-risk and low-risk populations, taking into account the practical limitations of NIPT services.

Synthetic data-driven machine learning models substantially improved predictive accuracy over the conventional *z*-score method. Whereas the *z*-score method exhibited decreasing sensitivity or PPV as prevalence declined, the LR models consistently maintained 100% performance, including real positive and synthetic negative samples. These performance changes are summarized in Table 5.

**Table 5.** Changes in sensitivity or PPV of *z*-score method and LR model with respect to prevalence level.

Category	*z*-score method	LR model
T13	77% to 64% (sensitivity)	100%
T18	98% to 68% (PPV)	100%
T21	98.9% to 70% (PPV)	100%

These results demonstrate that the synthetic data-based model maintains stable detection performance even when the evaluation data reflect actual prevalence levels. This also supports the methodological validity of using synthetic negatives as a substitute for real negative data under conditions of limited sample availability (Figs 9–12, available as supplementary data at *Bioinformatics Advances* online). Such an approach represents a practical improvement over the limitations of the *z*-score method and provides a strong foundation for enhancing the reliability and clinical utility of NIPT in the future.

Our methodology has two key innovative features, both driven by a synthetic data-driven approach. First, it enables the generation of sufficient synthetic positive and negative data (Figs 3 and 4, available as supplementary data at *Bioinformatics Advances* online), effectively addressing the lack of real positive cases in NIPT research. This provides a methodological novelty, as it allows virtually unlimited training and evaluation resources to be constructed with >99.9% similarity to real data. As a result, the LR-based AI model can detect subtle distinctions between positive and negative samples more sensitively and consistently than conventional *z*-score-based approaches. Second, by simultaneously considering variations in GC content, unique read count, and FF, our synthetic data-driven framework supports tailored detection optimized for the characteristics of each evaluation sample, thereby enhancing robustness and ensuring broad applicability across different experimental settings. Collectively, these innovations highlight the role of the synthetic data-driven paradigm in overcoming data scarcity and contribute a new framework to the body of knowledge in prenatal genomic analysis.

Nevertheless, the methodology presented in this study has the following three limitations. First, the accuracy of our method fundamentally depends on the quality of the synthetic data, with the FF being the most critical factor influencing its generation. Therefore, the accuracy of the FF directly determines the quality of the synthetic data, and high precision in FF estimation is essential for building a robust model. However, because our model was built using SNP-based FF estimates, errors were unavoidable when test samples calculated using Y chromosome-based FFs (Hudecova *et al.* 2014) or machine learning-based FFs (Kim et al. 2020) were applied (Figs 9–12, available as supplementary data at *Bioinformatics Advances* online).

Second, since the synthetic data were generated from 347 real male and 354 real female fetal samples, their characteristics inherently reflect the GC content and read count distribution of the original dataset. Therefore, if the GC content or read count distribution of new experimental data deviates significantly from that of the original real data, errors may occur. For this reason, it is essential to continuously monitor the characteristics of the input raw data in real time. If data that cannot be processed by the current model continue to occur, a new model should be built to reflect the characteristics of the updated data. In particular, if sample data are generated under the same experimental conditions but using a different sequencing platform, the GC profiles specific to that platform should first be examined, and reference datasets reconstructed by defining appropriate GC content distributions. Synthetic data can then be generated based on these updated distributions to establish a new model accordingly. Alternatively, if the current model is to be maintained, the in vitro process should be checked to ensure consistency with the original experimental conditions.

Third, as with conventional technologies, our approach also faces challenges in making accurate predictions in cases of fetal mosaicism. Since NIPT analyzes cell-free fetal DNA fragments derived from the placenta, discrepancies between placental and fetal cells or heterogeneity within the placental cell population—where some cells are negative and others are positive—can lead to detection errors (Van Opstal *et al.* 2016, Dhamankar *et al.* 2020). Our model also demonstrates near-perfect performance under standard conditions; however, when mosaicism is taken into account, the actual predictive accuracy may be somewhat reduced. Such reductions in accuracy may likewise occur in other NIPT-based approaches and require continued research to overcome this limitation.

## Supplementary Material

vbaf244_Supplementary_Data

## Data Availability

Source code for this study is available at https://github.com/genomecare-rnd/SyntheticData-NIPT.

## References

[vbaf244-B1] American College of Obstetricians and Gynecologists. Committee Opinion No. 640: cell-free DNA screening for fetal aneuploidy. Obstet Gynecol 2015;126:e31–7. 26287791 10.1097/AOG.0000000000001051

[vbaf244-B2] Bianchi DW , Parker RL, Wentworth J et al; CARE Study Group. DNA sequencing versus standard prenatal aneuploidy screening. N Engl J Med 2014;370:799–808. 24571752 10.1056/NEJMoa1311037

[vbaf244-B3] Dhamankar R , DiNonno W, Martin KA et al Fetal sex results of noninvasive prenatal testing and differences with ultrasonography. Obstet Gynecol 2020;135:1198–206. 32282607 10.1097/AOG.0000000000003791 PMC7170435

[vbaf244-B4] Goel N , Morris JK, Tucker D et al Trisomy 13 and 18—prevalence and mortality—a multi-registry population based analysis. Am J Med Genet A 2019;179:2382–92. 31566869 10.1002/ajmg.a.61365 PMC6848757

[vbaf244-B5] Hosmer DW , Lemeshow S, Sturdivant RX. Applied Logistic Regression, 3rd edn. Hoboken (NJ): Wiley, 2013.

[vbaf244-B6] Hudecova I , Sahota D, Heung MM et al Maternal plasma fetal DNA fractions in pregnancies with low and high risks for fetal chromosomal aneuploidies. PLoS One 2014;9:e88484. 24586333 10.1371/journal.pone.0088484 PMC3938419

[vbaf244-B7] Kim M , Kim JH, Kim K et al Cost-effective and accurate method of measuring fetal fraction using SNP imputation. Bioinformatics 2018;34:1086–91. 29126132 10.1093/bioinformatics/btx728

[vbaf244-B8] Kim S , Kim K, Jeon YJ. GenomomFF: cost-effective method to measure fetal fraction by adaptive multiple regression techniques with optimally selected autosomal chromosome regions. IEEE Access 2020;8:106880–8.

[vbaf244-B9] Liao C , Yin AH, Peng CF et al Noninvasive prenatal diagnosis of common aneuploidies by semiconductor sequencing. Proc Natl Acad Sci USA 2014;111:7415–20. 24799683 10.1073/pnas.1321997111 PMC4034209

[vbaf244-B10] Manzini G. An analysis of the burrows—wheeler transform. J ACM 2001;48:407–30.

[vbaf244-B11] Norton ME , Jacobsson B, Swamy GK et al Cell-free DNA analysis for noninvasive examination of trisomy. N Engl J Med 2015;372:1589–97. 25830321 10.1056/NEJMoa1407349

[vbaf244-B12] Presson AP , Partyka G, Jensen KM et al Current estimate of Down Syndrome population prevalence in the United States. J Pediatr 2013;163:1163–8. 23885965 10.1016/j.jpeds.2013.06.013 PMC4445685

[vbaf244-B13] Taylor-Phillips S , Freeman K, Geppert J et al Accuracy of non-invasive prenatal testing using cell-free DNA for detection of Down, Edwards and Patau syndromes: a systematic review and meta-analysis. BMJ Open 2016;6:e010002. 10.1136/bmjopen-2015-010002 PMC4735304 26781507

[vbaf244-B14] Van Opstal D , Srebniak MI, Polak J et al False negative NIPT results: risk figures for chromosomes 13, 18 and 21 based on chorionic villi results in 5967 cases and literature review. PLoS One 2016;11:e0146794. 26771677 10.1371/journal.pone.0146794 PMC4714811

[vbaf244-B15] Zhang H , Gao Y, Jiang F et al Non-invasive prenatal testing for trisomies 21, 18 and 13: clinical experience from 146,958 pregnancies. Ultrasound Obstet Gynecol 2015;45:530–8. 25598039 10.1002/uog.14792

