# Supplementary material for: Synthetic data-driven AI approach for fetal chromosomal aneuploidies detection
Source: Bioinform Adv. 2025 Oct 6;5(1):vbaf244. doi: 10.1093/bioadv/vbaf244 (PMC12557104; doi:10.1093/bioadv/vbaf244)
Supplement: vbaf244_Supplementary_Data [file vbaf244_supplementary_data.docx]

# SUPPLEMENTARY INFORMATION

# Manuscript Title: Synthetic data-driven AI approach for fetal chromosomal aneuploidies detection

# Authors: Changhoe Hwang, Krishna Prasad Adhikari, Gyeongin Oh and Sunshin Kim

# CORRELATION BETWEEN REAL DATA ANDSYNTHETIC DATA


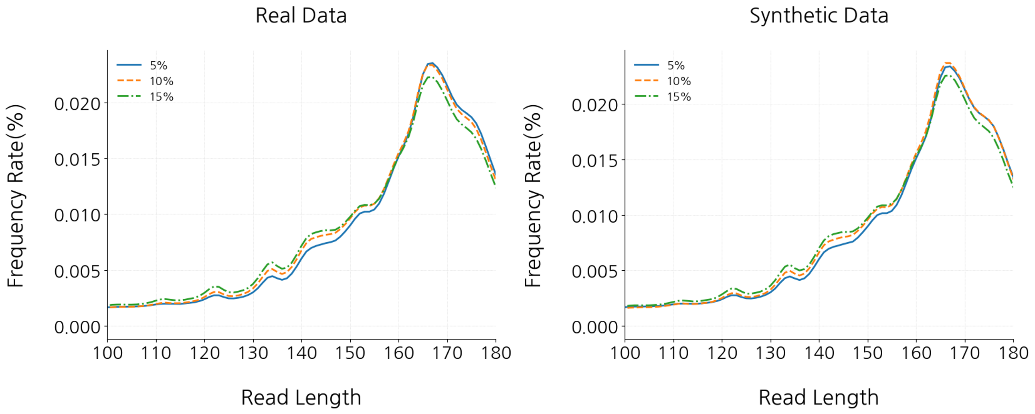


Figure S1. Correlation graphs of read length frequency distributions between real and synthetic Data

# DISTRIBUTION OF EVALUATION SAMPLES FOR ACA DETECTION BY Z-SCORE METHOD

In the scatter plots shown in Sections 2, 3, and 5, the black dots representing 701 samples correspond to reference data used both as the source for generating synthetic data for autosomes and as the baseline reference set for z-score–based autosomal chromosome aneuploidy (ACA) classification. These data consisted of 347 negative (normal) male fetal samples and 354 negative female fetal samples, from which 242 male and 249 female samples were selected to generate 160,000 synthetic positive (abnormal) and negative training samples. The reference set for z-score method classification consisted of 701 negative samples after outlier exclusion. In addition, 97 male and 96 female fetal samples not used in training were used to generate 300,000 synthetic positive and negative evaluation samples. Each plot displays the normalized read count of the target chromosome — defined as $Normalized Read Count=\frac{Read count of target chromosome \times3,000,000 (3M)}{Total sample read count}$ — plotted against GC content (%).


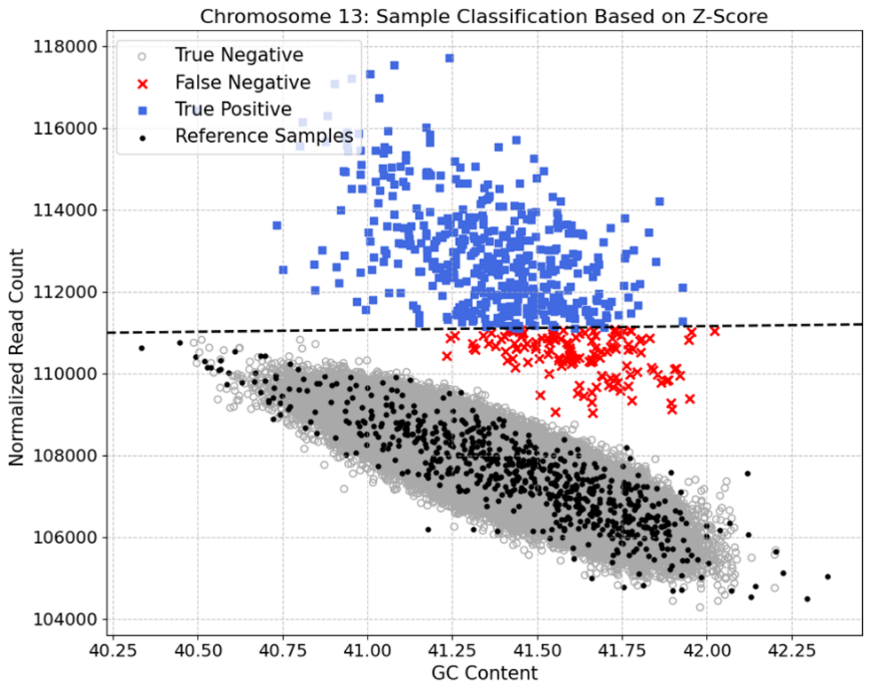


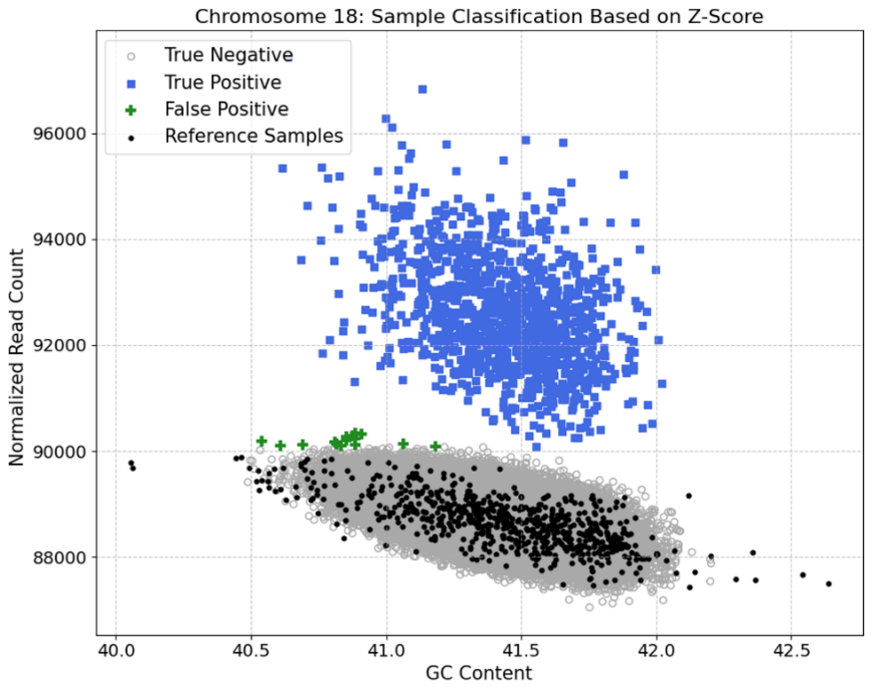


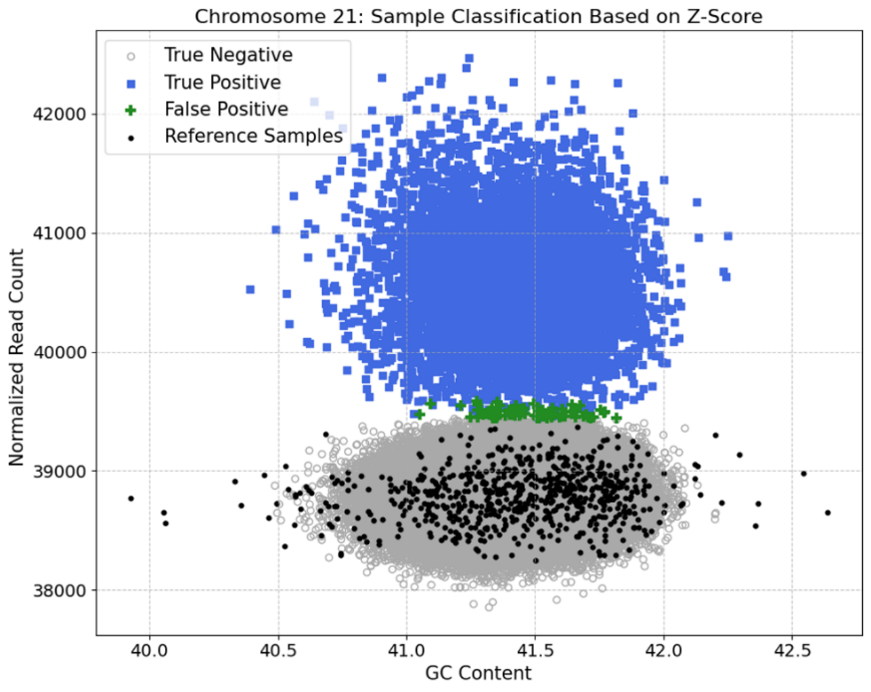


**Figure S2.** Evaluation sample distribution by z-score – After generating 300,000 synthetic negative samples, statistical outliers were removed, and the data were upscaled by up to 40 times based on prevalence. For T13 (trisomy 13), the final dataset included 268,549 negative samples and 594 positive samples. For T18 (trisomy 18), there were 276,076 negative samples and 1,192 positive samples. For T21 (trisomy 21), the dataset consisted of 238,126 negative samples and 7,912 positive samples.

Excluding the reference data, the remaining samples—classified as TN (true negative), FN (false negative), TP (true positive), and FP (false positive)—were evaluation data synthesized by randomly combining three non-overlapping samples from 97 real negative male and 96 real negative female fetal datasets. These samples served to visually evaluate the classification performance of the model.

Figure S2 shows that, in the case of T13, positive samples (TP and FN) tend to cluster in regions with high read counts. However, the boundary between positive and negative samples is not clearly separated, and the two form closely packed, nearly overlapping regions. Notably, some positive samples were classified as FN despite being located near other TP samples, indicating that the z-score method failed to sufficiently capture positive cases within that region.

In contrast, for T18 and T21, FP were observed in certain regions where negative samples overlapped with the distribution of positive samples. This indicates that the z-score–based classification method failed to effectively distinguish negative samples located within the negative sample distribution from positive ones, which may contribute to a decrease in PPV.

**Table S1.** z-score method classification results with evaluation samples for trisomy 13, 18, and 21

| **Chromosome** | **Reference Data (Black dot)** | **Negative Samples** | **Positive Samples** | **TP** | **TN** | **FP** | **FN** |
| --- | --- | --- | --- | --- | --- | --- | --- |
| 13 |  | 268,477 | 596 | 458 | 268,549 | 0 | 138 |
| 18 | 691 | 276,020 | 1,189 | 1,189 | 276,005 | 15 | 0 |
| 21 |  | 238,064 | 7,908 | 7,912 | 237,984 | 80 | 0 |

# DISTRIBUTION OF TRAINING SYNTHETIC SAMPLES: TRISOMY 13/18/21


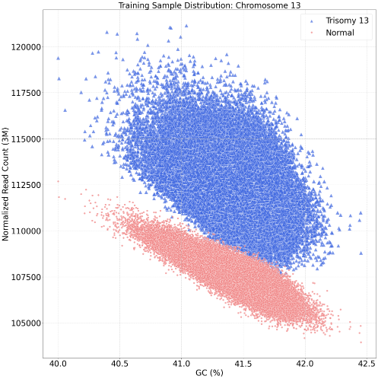


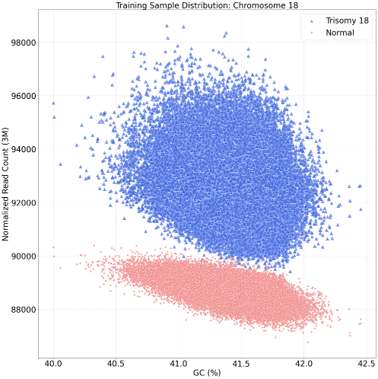


**
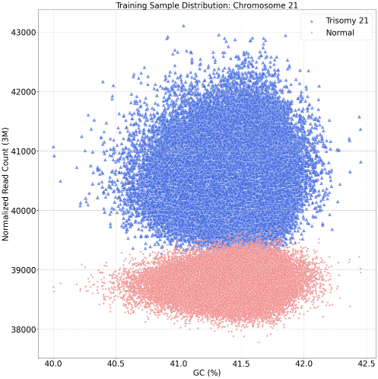
**

Figure S3. Training sample distribution: chromosomes 13/18/21

Figure S3 presents scatter plots visualizing the distribution of training data for T13, T18, and T21. In each plot, trisomy positive samples are marked with blue triangles, while negative samples are represented by red dots.

The distribution reflects 80,000 synthetic positive and 80,000 synthetic negative samples (160,000 in total) generated from 242 male and 249 female negative fetal samples. To enhance model robustness, the final dataset used for logistic regression (LR) training underwent quality refinement by removing outliers based on GC content, fetal fraction (FF), and target chromosome read counts, thereby ensuring clearer class separation.

The distribution reveals a consistent distinction between negative and positive samples, providing a foundation for the model to learn class boundaries based on these differences. The 3D scatter plots below, based on GC content, FF, and target chromosome read count, clearly show this separation.


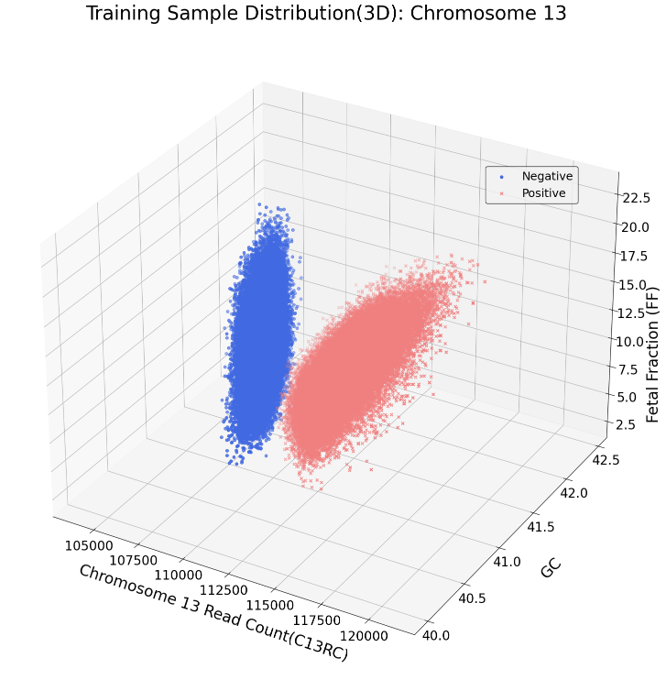


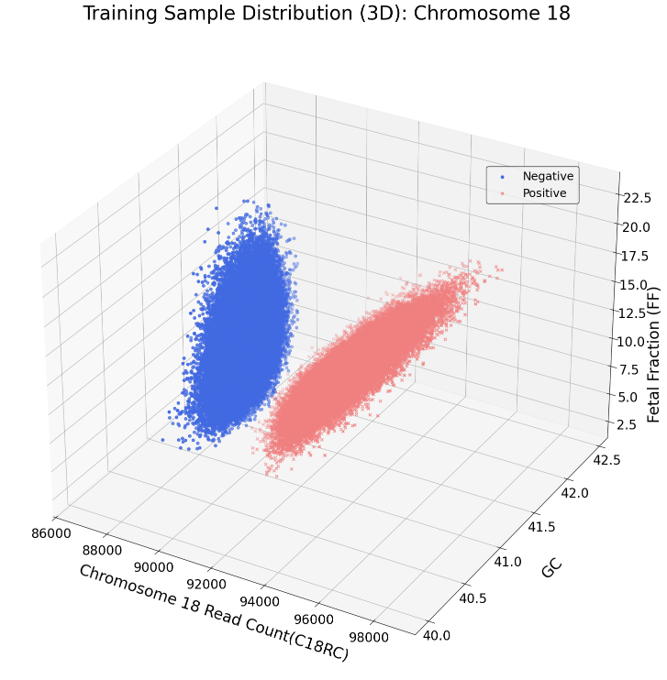

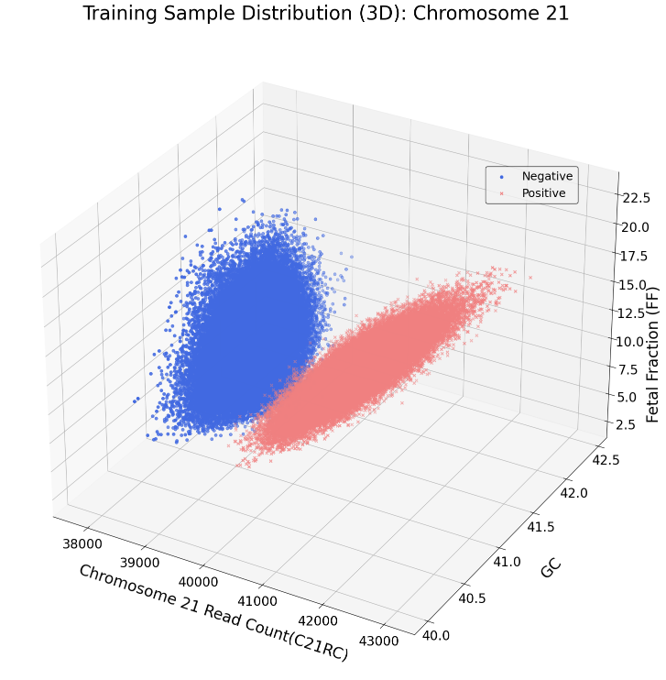


Figure S4. Training Sample Distribution (3D): Chromosomes 13/18/21

# ACA DETECTING PERFORMANCE EVALUATION WITH PREVALENCE

## **- Real Positive / Synthetic Negative Samples**

### **4.1. Trisomy 13**

**
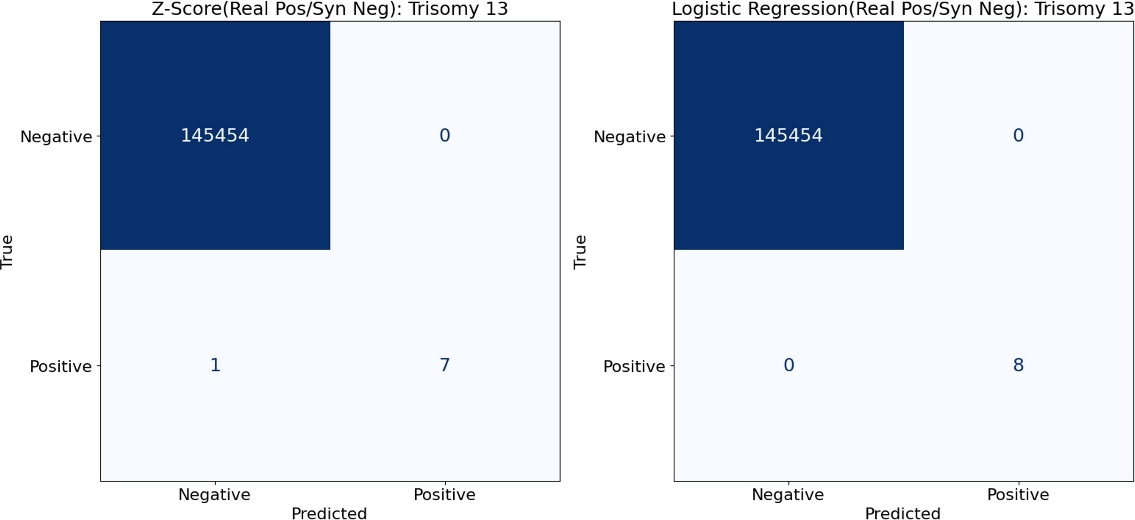
**

Figure S5. Confusion matrices for trisomy 13 detection using prevalence samples: z-score and logistic regression

The z-score method demonstrated high specificity and PPV without FPs, but its sensitivity remained relatively low at 87.5%. In contrast, the LR model achieved perfect performance across all metrics, indicating its exceptional classification accuracy on the T13 dataset.

Table S2. Comparative performance of z-score and logistic regression for trisomy 13 detection with prevalence samples

| **Model** | **Sensitivity** | **Specificity** | **PPV** | **NPV** |
| --- | --- | --- | --- | --- |
| Z-score | 0.875 | 1.0 | 1.0 | 0.999993 |
| Logistic regression | 1.0 | 1.0 | 1.0 | 1.0 |

### **4.2. Trisomy 18**


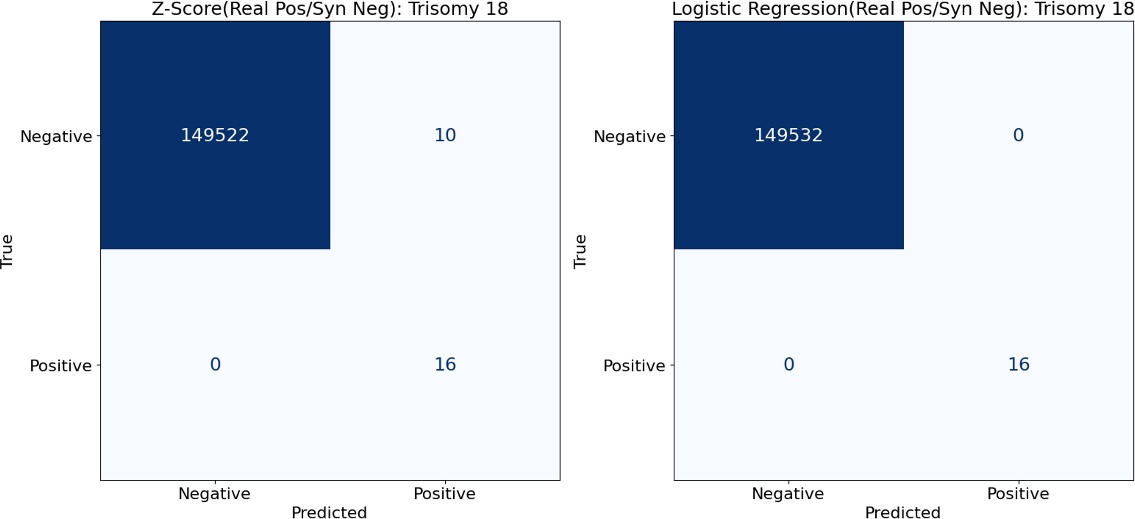


Figure S6. Confusion matrices for trisomy 18 detection using prevalence samples: z-score and logistic regression

Table S3. Comparative performance of z-score and logistic regression for trisomy 13 detection with prevalence samples

| **Model** | **Sensitivity** | **Specificity** | **PPV** | **NPV** |
| --- | --- | --- | --- | --- |
| Z-score | 1.0 | 0.999933 | 0.615385 | 1.0 |
| Logistic regression | 1.0 | 1.0 | 1.0 | 1.0 |

In the classification performance for T18, the z-score method achieved 100% sensitivity but showed a low PPV of 61.5%, indicating a high rate of FPs. In contrast, the LR model demonstrated perfect performance across all evaluation metrics for T18 as well, confirming its capability for highly reliable classification.

### **Trisomy 21**


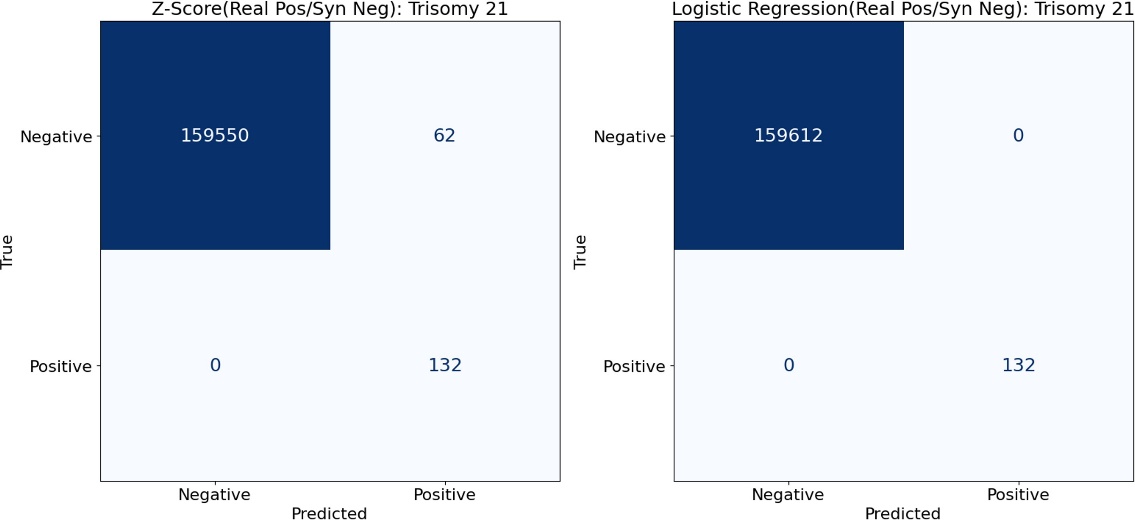


Figure S7. Confusion matrices for trisomy 21 detection using prevalence samples: z-score and logistic regression

Table S4. Comparative performance of z-score and logistic regression for trisomy 21 detection with prevalence samples

| **Model** | **Sensitivity** | **Specificity** | **PPV** | **NPV** |
| --- | --- | --- | --- | --- |
| Z-score | 1.0 | 0.999612 | 0.680412 | 1.0 |
| Logistic regression | 1.0 | 1.0 | 1.0 | 1.0 |

For T21, the z-score method maintained high sensitivity, but its PPV was approximately 68%, indicating a considerable number of FPs. The LR model also achieved perfect performance in this classification assessment, establishing itself as the most effective approach for detecting T21.

In summary, the LR models consistently outperformed the z-score method across T13, T18, and T21, demonstrating superior and stable performance—particularly by achieving high PPV without FPs. Notably, the z-score classifier consistently produced FPs across chromosomes 18 and 21, and in the case of chromosome 13, A FN also occurred, leading to a decline in sensitivity. This trend aligns with the performance degradation previously observed in the synthetic data results. Additionally, by evaluating the model using synthetic negative data alongside real positive samples, this study was able to indirectly validate that the synthetic data reasonably reflect the statistical characteristics of real negative data. This provides meaningful evidence supporting the utility of synthetic data for constructing evaluation environments and validating models under resource-constrained conditions.

### **Distribution of Prevalence Samples for Trisomy Detection by Z-Score Method**


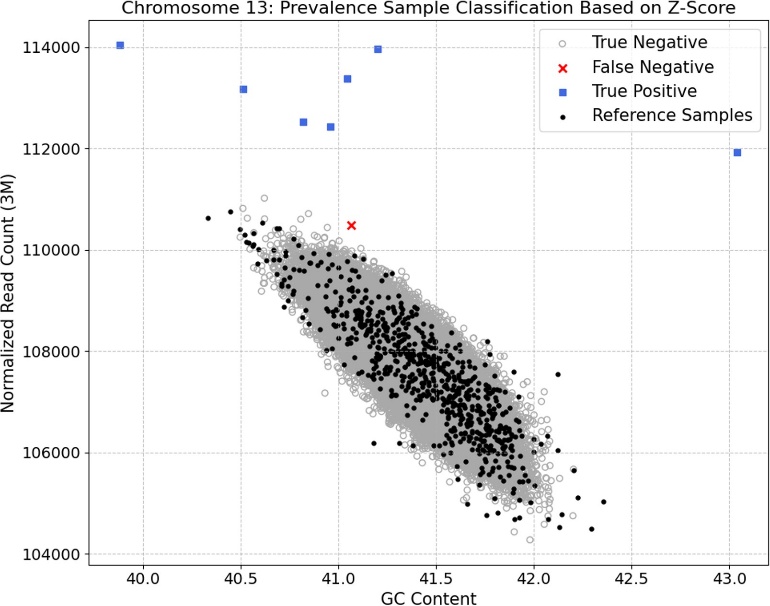


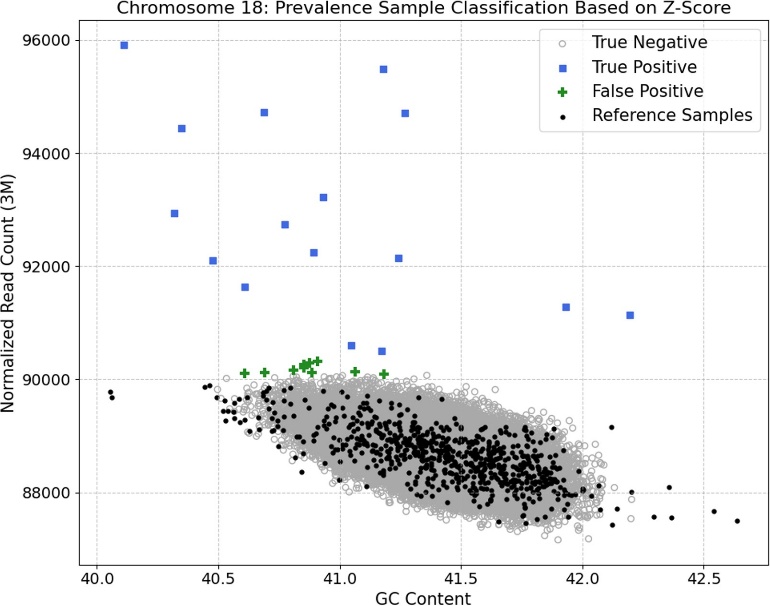


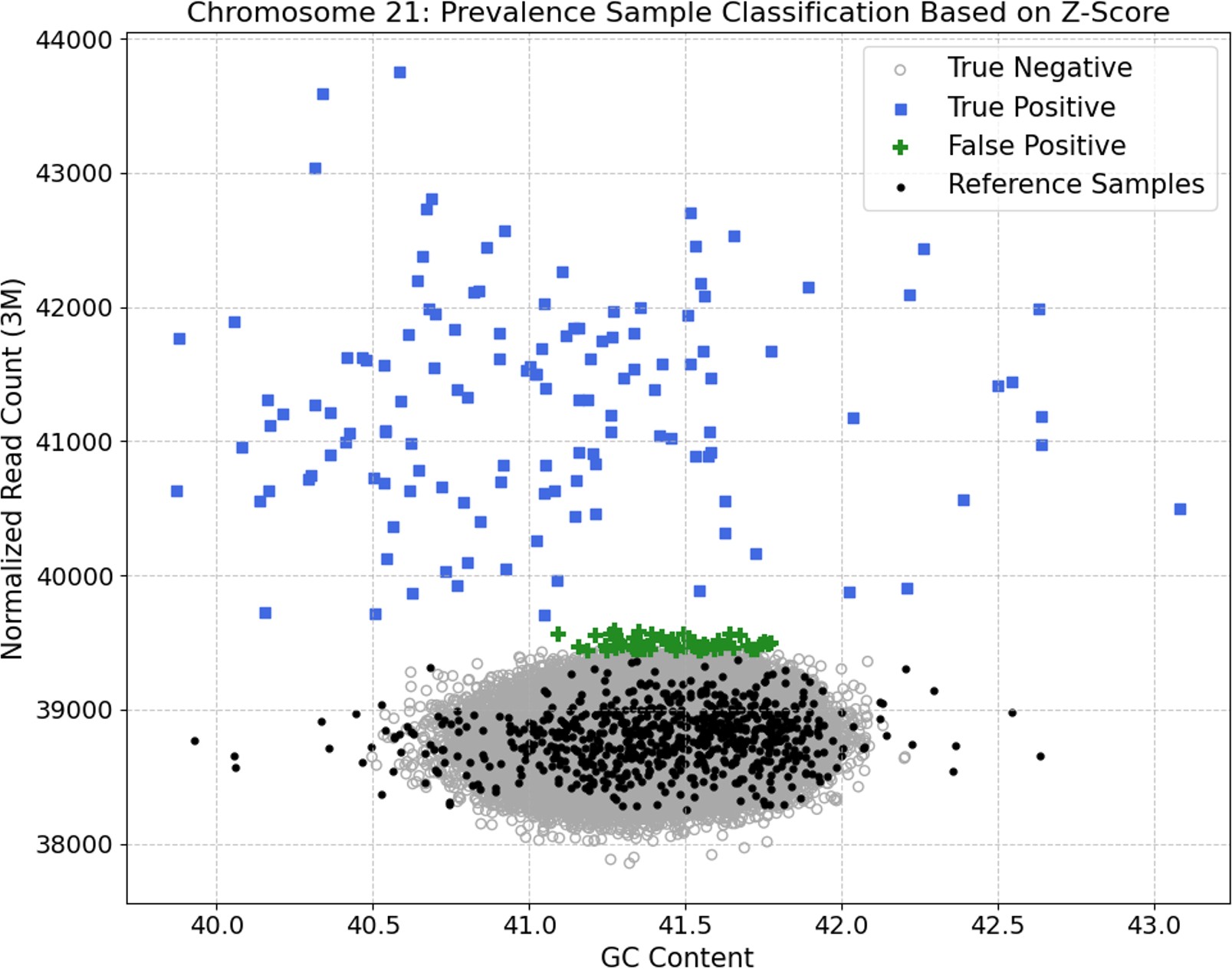


Figure S8. Prevalence sample distribution by z-score: chromosomes 13/18/21

This figure visualizes the classification results of the prevalence samples for T13, T18, and T21 using the z-score method. Each scatter plot is constructed based on GC content and normalized read count, illustrating the relative distribution of TP, TN, FP, FN, and reference samples.

In the case of T13, one FN sample was observed, which appears to result from the ambiguity in the decision boundary between positive and negative samples. For both T18 and T21, the distributions of TP samples were generally well separated; however, a substantial number of FP samples appeared in regions adjacent to the normal data distribution.

Across all three chromosomes, the z-score method classification repeatedly misclassified samples in regions overlapping with the boundary of the normal data distribution, and this pattern was consistently observed in the prevalence evaluation.

# ACA DETECTION PERFORMANCE EVALUATION WITH REAL SAMPLES

## **- Real Positive / Real Negative Samples**

To evaluate the classification performance of the model trained on synthetic data using real (clinical) data, we conducted an assessment using only real positive and negative samples. This approach provides a key advantage in that it enables direct validation of how well the synthetic data–based model performs in actual clinical settings.

To ensure the validity of this evaluation, we restricted the clinical samples to those meeting specific quality criteria: GC content between 39.5% and 43%, and total read count exceeding 2 million. In addition, read counts for target chromosomes were normalized to a fixed total of 3 million reads, and only samples with an FF of 8% or higher were included in the analysis. To obtain a sufficient number of real negative samples, we estimated FF using a Y chromosome–based method (Hudecova et al., 2014) for male fetuses, and a machine learning–based method (Kim et al., 2020) using autosomal chromosomes for female fetuses. We applied a threshold of 8% FF because the LR model was developed using SNP-based FF estimates, whereas the clinical samples used in this evaluation employed alternative methods based on Y chromosomes or autosomal signals. At lower FF levels, discrepancies between these estimation methods can also introduce noise variance that obscures the true FF, making the values less reliable for evaluation.

### **5.1. Trisomy 13**


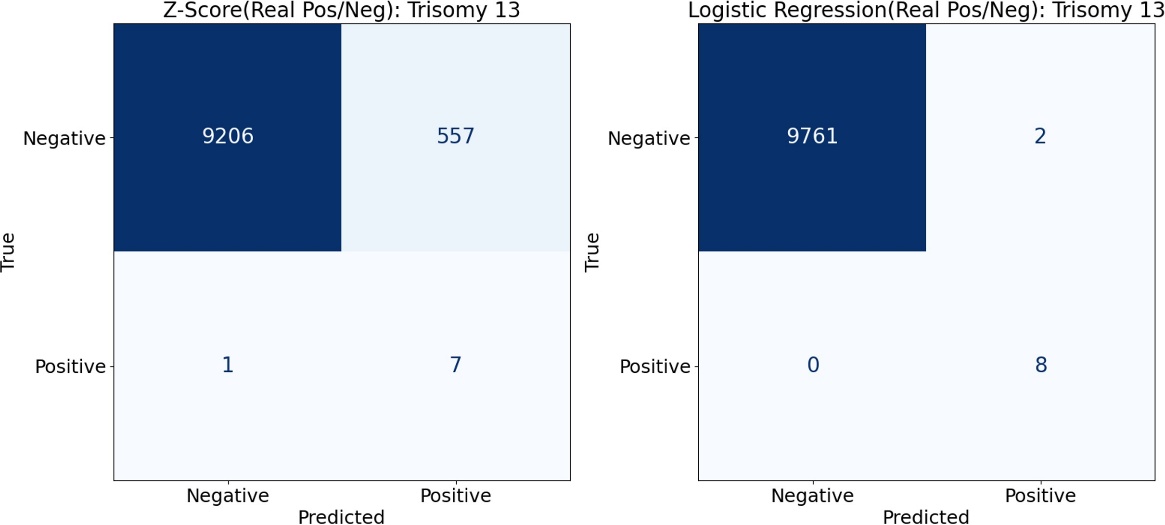


Figure S9. Confusion matrices for trisomy 13 detection using real samples: z-score and logistic regression

The z-score–based method exhibited poor performance in classifying real T13 samples, with a sensitivity of 87.5% and a PPV of only 1.2%. Notably, it produced 557 FPs, indicating that the vast majority of predicted positives were actually negative samples. In addition, one FN case was observed, suggesting that the method also failed to capture all true positives. In contrast, the LR model achieved 100% sensitivity, and a PPV of 80%, consistently outperforming the z-score method across all metrics. These results suggest that the LR model provides a far more stable and reliable approach for T13 detection in real clinical settings.

### **5.2. Trisomy 18**


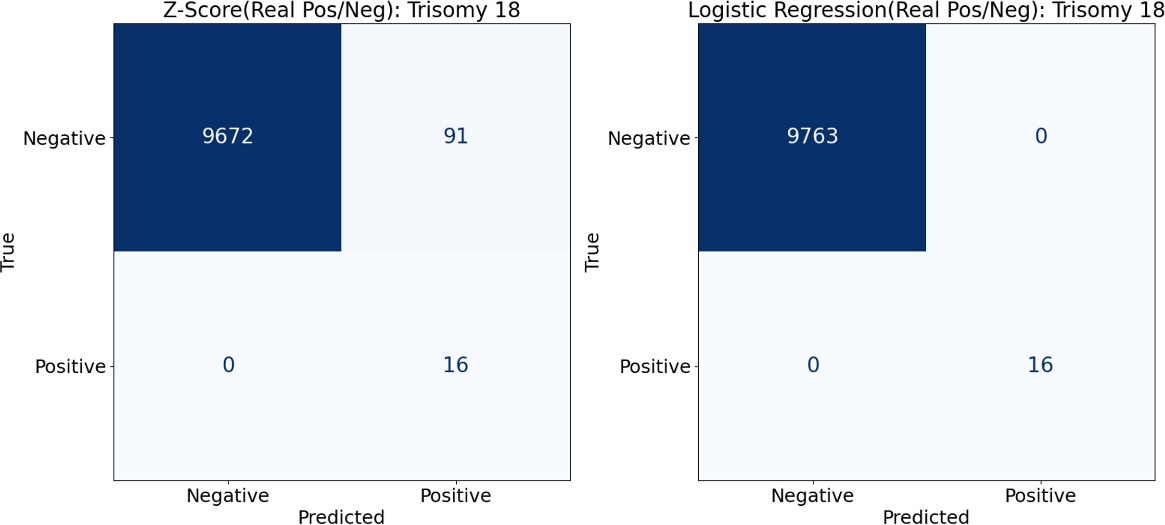


Figure S10. Confusion matrices for trisomy 18 detection using real samples: z-score and logistic regression

In the real-data-based evaluation for T18, the z-score method achieved 100% sensitivity, successfully detecting all positive samples. However, 91 FPs were observed, resulting in a reduced specificity of 99.1% and a notably low PPV of 15%. This indicates that a substantial number of negative samples were incorrectly classified as positives. In contrast, the LR model accurately classified all samples, achieving perfect performance across all metrics, including sensitivity and PPV. These findings clearly demonstrate that LR outperforms the z-score method in the detection of T18.

### **Trisomy 21**


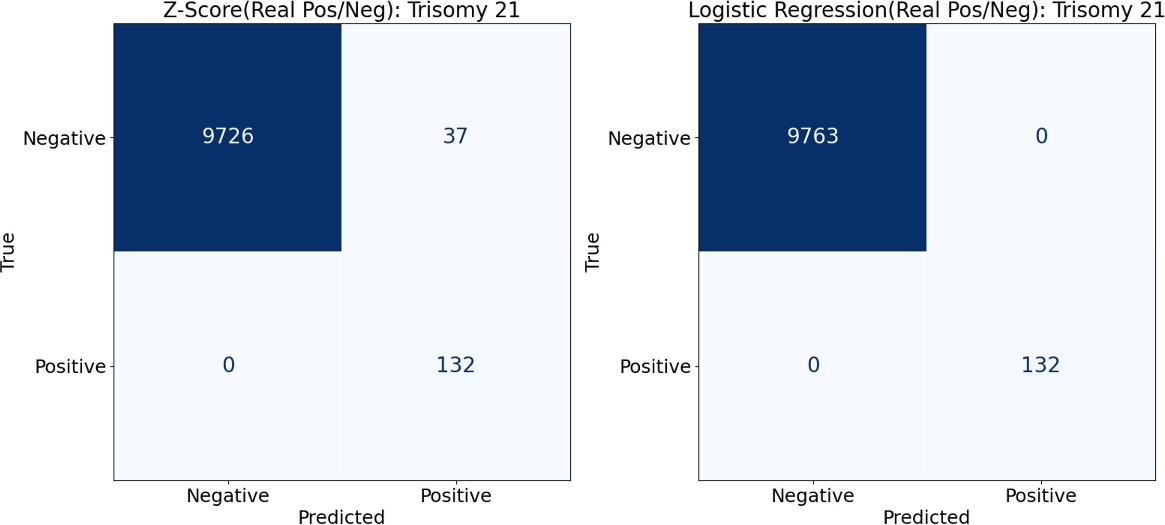


Figure S11. Confusion matrices for trisomy 21 detection using real samples: z-score and logistic regression

In T21 detection, the z-score–based method maintained high sensitivity; however, it misclassified a substantial number of negative samples as positive, resulting in reduced specificity (99.6%) and PPV (78.1%). This reflects a considerable number of FPs and highlights the potential risk of overdiagnosis in a screening context.

In contrast, the LR model achieved perfect scores across all performance metrics for T21 as well, demonstrating its high reliability and applicability under real clinical conditions.

### **Distribution of Real Samples for Trisomy Detection by Z-Score Method**


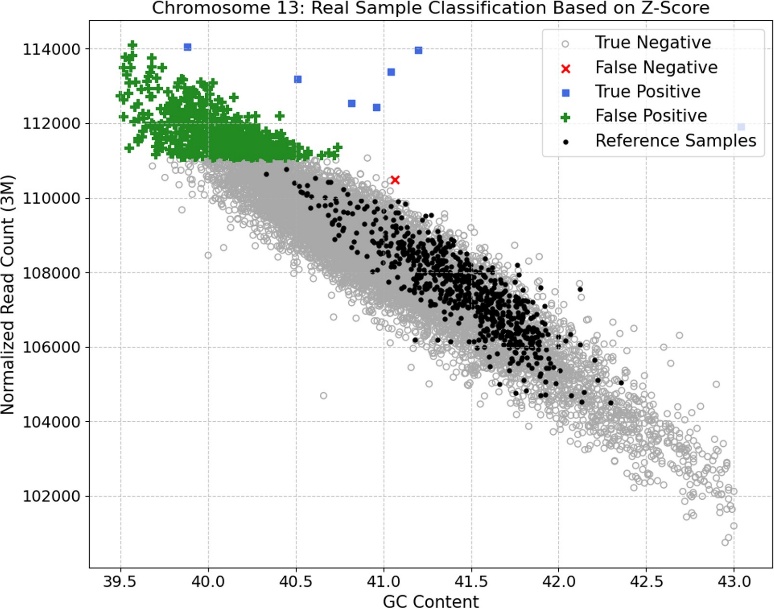


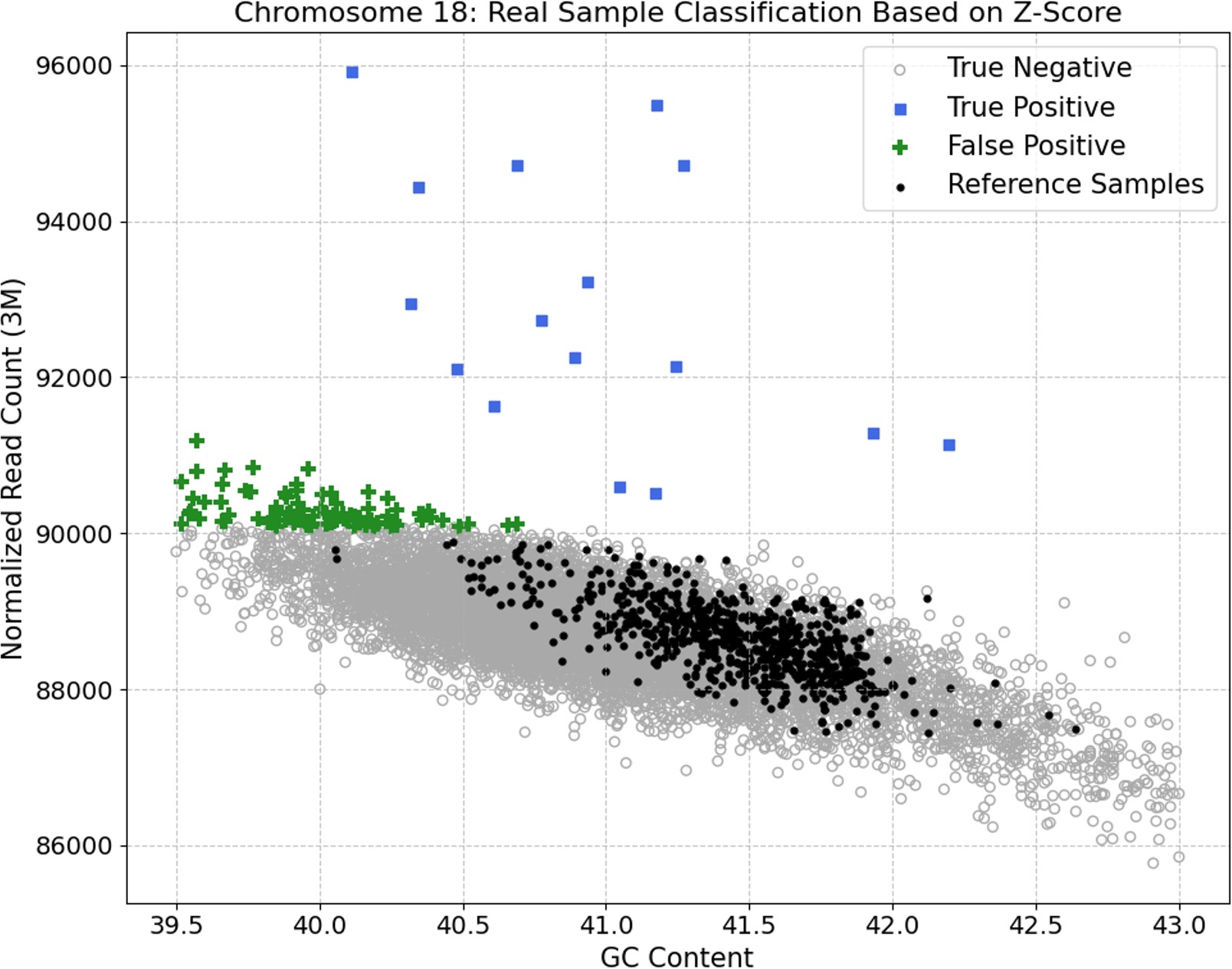


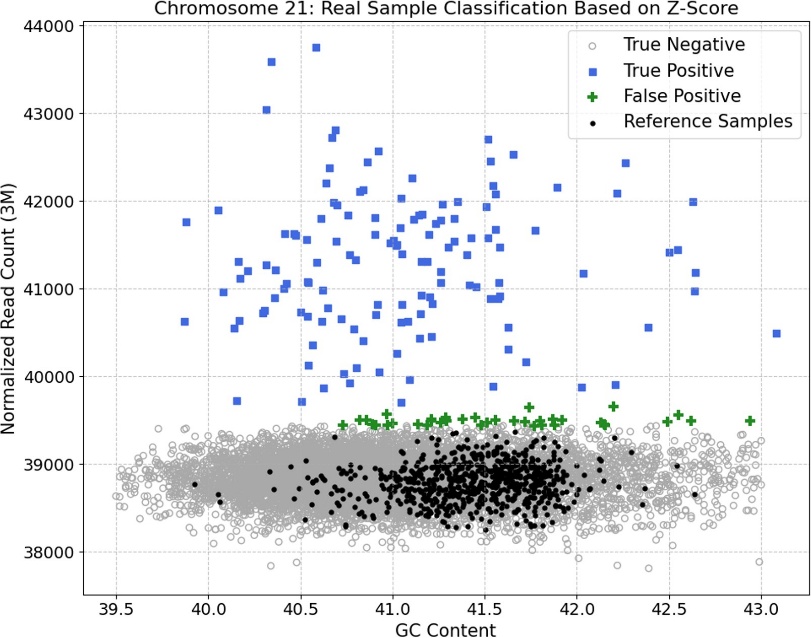


Figure S12. Real sample distribution by z-score: chromosomes 13/18/21

Figure S12 visualizes the classification results of T13, T18, and T21 based on real (clinical) data using the z-score method. Each scatter plot shows the distribution of classified samples according to GC content and normalized read count (fixed at 3 million).

For T13, TP samples were primarily located in regions with high read counts; however, a large number of FPs overlapped with the distribution of negative samples, leading to frequent misclassifications. Additionally, one FN case was observed, consistent with previous results, indicating that the z-score method failed to correctly identify some positive cases. In the case of T18, the TP and TN samples were relatively well separated, but some FP samples appeared near the boundary of the normal data distribution, resulting in actual misclassification. For T21, a significant number of FP samples were located within the dense region of negative samples, clearly illustrating the tendency of the z-score method to overclassify negatives as positives.

Taken together, these results show that in real data–based z-score classification, FPs repeatedly occur in regions overlapping with the normal data distribution, and for T13, a FN was also present—indicating an overall lack of classification stability. Such misclassification patterns were consistently observed in both T18 and T21, regardless of the evaluation dataset, revealing a structural limitation inherent to the z-score–based classification approach.

# SCA DETECTION PERFORMANCE EVALUATION WITH REAL SAMPLES


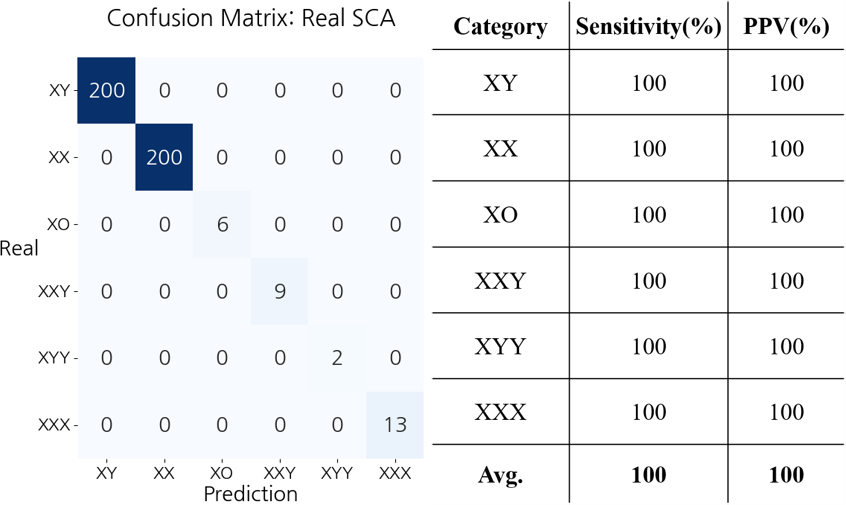


Figure S13. Performance of SCA detection model about real data

Additionally, Figure S13 presents the results of applying the SCA model developed in this study to real data. As shown, the LR model trained using the proposed synthetic data generation methodology accurately classified all samples, achieving 100% performance for both negative (XX, XY) and positive (XO, XXY, XYY, XXX) cases.

# ALGORITHMS

The process of synthetic data generation and model training described in the manuscript is visualized in the following algorithmic flow chart and is also available at <https://github.com/genomecare-rnd/SyntheticData-NIPT>.


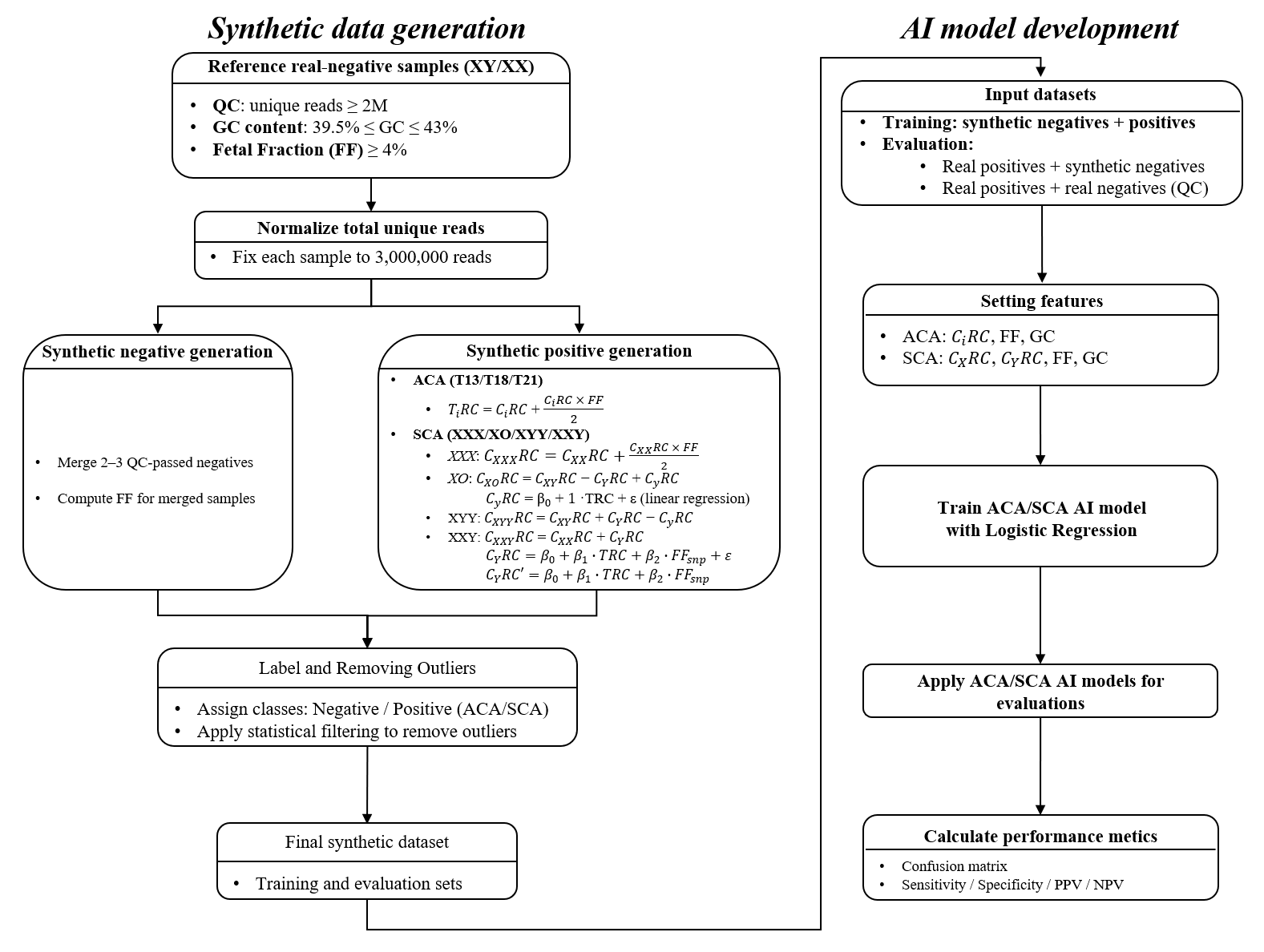


Figure S14. Algorithm flow of synthetic data generation and AI model development
